# Hyperglycemia- induced innate immune tolerance involves the metabolic and epigenetic rewiring in human alveolar macrophages

**DOI:** 10.3389/fimmu.2026.1834572

**Published:** 2026-05-07

**Authors:** Jiang Wang, Na Yuan, Bin Wang, YongHui Qiu, Bo Wang, LiXin Xie

**Affiliations:** 1 Senior Department of Pulmonary and Critical Care Medicine, Chinese PLA General Hospital, Beijing, China; 2 Medical School of Chinese PLA, Beijing, China; 3 Department of Pulmonary & Critical Care Medicine, the First Medical Center of Chinese PLA General Hospital, Beijing, China; 4 Department of Thoracic Surgery, the First Medical Center of Chinese PLA General Hospital, Beijing, China; 5 Department of Thoracic Surgery, the Fourth Medical Center of Chinese PLA General Hospital, Beijing, China

**Keywords:** alveolar macrophages, epigenetic modification, hyperglycemia, immunological tolerance, innate immune memory, metabolic reprogramming

## Abstract

**Introduction:**

Diabetic patients have increased susceptibility to pulmonary infections. However, whether hyperglycemia contributes to this susceptibility via the immune memory of alveolar macrophages (AMs) remains unclear.

**Methods:**

Primary human AMs from patients with diabetes were isolated. An *in vitro* hyperglycemia-induced immune memory model was established. Immune memory phenotypes were assessed by cytokine secretion and phagocytosis upon secondary stimulation. Metabolic profiles were analyzed by Seahorse and LC-MS metabolomics. Transcriptional and epigenetic reprogramming were examined using RNA-seq, ATAC-seq, and CUT&Tag for H3K4me3.

**Results:**

AMs exposed to hyperglycemia, either *in vitro* or derived from diabetic patients, exhibited a persistent immune-tolerant phenotype *in vitro* with reduced pro-inflammatory cytokines and impaired phagocytosis. Metabolically, tolerant AMs showed a decrease in oxidative phosphorylation with no compensatory increase in glycolysis, accompanied by reprogramming of lipid metabolism program (acylcarnitine accumulation and downregulation of membrane lipids). Transcriptional analysis revealed downregulation of genes involved in inflammation and upregulation of autophagy and apoptosis-related genes. Epigenetically, tolerant AMs showed an association with increased chromatin accessibility and enhanced H3K4me3 modification at the regulatory regions of autophagy and apoptosis-related genes.

**Discussion:**

Hyperglycemia induces immune tolerance in human AMs through metabolic reprogramming (impaired fatty acid oxidation, lipid dysregulation) and epigenetic modifications at regulatory regions of autophagy and apoptosis-related genes. These findings suggest a potential mechanistic link between hyperglycemia and increased pulmonary infection susceptibility in diabetic patients, and identify candidate immunomodulatory targets for further investigation.

## 1 Introduction

Diabetes mellitus, a chronic metabolic disorder characterized by persistent hyperglycemia, substantially impairs quality of life and increases mortality risk (1–3). In 2021, the number of diabetic patients in China exceeded 118 million, accounting for approximately 22% of the global diabetic population, which has become a significant global public health challenge (4). Beyond its role in metabolic dysregulation, diabetes mellitus is a major risk factor for numerous complications, particularly cardiovascular diseases (CVDs) and infections (5, 6), which has drawn considerable attention. Compared to non-diabetic individuals, diabetic patients exhibit a significantly higher risk of developing pulmonary infections, further exacerbating organ damage and leading to higher mortality in advanced stages (7, 8). Despite advances in clinical management strategies, including the use of antibiotics and supportive care, this situation has not been effectively reversed. Therefore, elucidating the link between increased susceptibility to pulmonary infection and aberrant pulmonary immune responses in diabetic patients is of great importance. Immune memory has long been considered a hallmark of adaptive immunity, confined to B cells and antigen-specific T cells (9). This involves the establishment of memory upon initial antigen (pathogen) exposure, enabling a more rapid and robust protective response upon re-stimulation with the same pathogen (10). However, the concept and subsequent research on “trained immunity” have challenged this classical immunological paradigm (11, 12). Following initial encounter with specific stimuli, such as pathogen-associated molecular patterns or pathogens, innate immune cells undergo distinct functional adaptations, exhibiting either enhanced (trained immunity) or suppressed (tolerance) responses upon secondary stimulation (13). After the initial stimulus is removed, the functional immune state in cells with immune memory typically reverts to baseline levels (14). These cells commonly undergo metabolic reprogramming and chromatin remodeling, characterized by alterations in oxidative phosphorylation (OXPHOS), glycolysis, fatty acid metabolism, and alterations in active epigenetic histone marks located on promoters and distal enhancers (15, 16). Trained immunity enhances rapid and effective non-specific responses against secondary challenges and thus is usually considered beneficial for host defense. In contrast, immunological tolerance can serve as a negative regulatory mechanism crucial for preventing excessive inflammatory damage, mitigating the detrimental effects of infection on host health (17). However, if inappropriately induced in some cases (e.g., sepsis), it may lead to diminished or even exhausted immune defense (18). Understanding how specific metabolic signals and epigenetic mechanisms contribute to the transition of macrophages towards a tolerant state is a frontier area in innate immune memory research. Macrophages are a primary focus of immune memory research, with increasing attention in recent years on tissue-resident macrophages. As the most abundant innate immune cell population residing in the lungs, alveolar macrophages (AMs) constitute the first line of defense within the alveoli and airways. They not only orchestrate the initiation and resolution of pulmonary immune responses but also perform non-immune, tissue-specific, and homeostatic functions (19, 20). The role of hyperglycemia as an initiating stimulus in inducing trained immunity-termed “hyperglycemic memory”— has been explored in various cell types, including peripheral blood mononuclear cells, bone marrow-derived macrophages (BMDMs), and atherosclerotic plaque macrophages (21). However, whether AMs, as resident macrophages with significant functional plasticity, exhibit a distinct immune memory profile upon high glucose pre-stimulation rather than a pro-inflammatory trained immunity phenotype in monocyte-derived macrophages, is a central focus of this study and has not been previously attempted. Furthermore, research on innate immune memory in macrophages has largely relied on immortalized cell lines, mouse AMs, or BMDMs, which lack the long-term shaping of the alveolar microenvironment (e.g., low glucose, high oxygen, lipid-rich). These limitations hinder accurate recapitulation of the unique phenotype and function of human tissue-resident AMs. Our study directly utilizes AMs isolated from human lung tissue, which better preserve their original tissue-specific characteristics and may more accurately reflect human pathophysiological responses.

We hypothesize that immunological tolerance in AMs induced by elevated glucose levels contributes to the increased susceptibility to pulmonary infections in diabetic patients. Therefore, this study aims to investigate whether hyperglycemia induces disease-related functional alterations in human AMs and whether these changes persist after glucose normalization, constituting immune memory. On this basis, using cellular functional assays, metabolomics, transcriptomics, and epigenomics, we will examine whether hyperglycemia induces a tolerant phenotype in AMs and analyze the underlying mechanisms. This research provides a theoretical basis for understanding the host immune response in diabetic patients with secondary pulmonary infections and for exploring immunomodulatory therapies to reverse the tolerant state.

## 2 Materials and methods

### 2.1 Human tissue collection and processing

This study involving human tissues was conducted in accordance with the Declaration of Helsinki and received ethical approval from the Ethics Committee of the General Hospital of the People’s Liberation Army of China (Ethics No. S2020-430-01). Written informed consent was obtained from all patients or their legal representatives. The fresh macroscopically normal lung tissues from lobectomy were obtained from patients undergoing lobectomy for pulmonary nodules or masses in the Department of Thoracic Surgery affiliated with Chinese PLA General Hospital. Tissue specimens macroscopically free of tumor were excised at least 2 cm from the lesion margin and near the edge of the lobe, ensuring no interference with postoperative pathological diagnosis (22). Fresh tissues were immediately immersed in cold tissue storage solution (Miltenyi, Cat# 130-100-008, Germany) and transported to the laboratory for cell isolation. We acknowledge that despite macroscopically normal appearance, the tissue microenvironment may still be influenced by the adjacent tumor or systemic patient factors.

Inclusion criteria were as follows: patients with nodules or masses undergoing lobectomy, with availability of sufficient macroscopically normal lung tissue from lobectomy tissues for cell isolation. Patients were assigned to two groups: one with diabetes (diabetic) group and another without diabetes (control) group. Exclusion criteria comprised: (1) age < 18 years; (2) gross tumor invasion of the bronchus, multiple intrapulmonary metastases, or ill−defined tumor margins; (3) concomitant respiratory diseases affecting lung tissue architecture, including asthma, chronic obstructive pulmonary disease, pulmonary fibrosis, bronchiectasis, or active pulmonary infection; (4) systemic diseases affecting immune function; (5) prior neoadjuvant chemotherapy, radiotherapy, or systemic immunotherapy. All clinical information was obtained from patients’ medical records. The **Supplementary Table 1** summarizes the clinical characteristics of the patients.

### 2.2 Alveolar macrophages isolation

Lung tissue specimens were minced with a sterile scalpel. The tissue blocks were gently washed in Hanks’ Balanced Salt Solution (HBSS) (Solarbio, Cat# H1147, China) using sterile forceps. The wash fluid was passed through a 70 μm cell strainer (Corning, Cat# CLS431751, USA) and centrifuged for 10 minutes (300×g, 10min). Cells were washed in 50 mL cold HBSS three times and then resuspended in sterile glucose-free RPMI 1640 medium (Thermo Fisher Scientific, Cat# 11879020, USA). This medium was supplemented with 10% heat-inactivated fetal bovine serum (Gibco, Cat# 10091148, USA), 1% HEPES (Gibco, Cat# 15630130, USA), 1mM sodium pyruvate (Gibco, Cat# 11360070, USA), penicillin 100 U/ml and streptomycin 100 µg/ml (Gibco, Cat# 15140122, USA), and varying concentrations of glucose (0.6 mM, 5 mM, or 20 mM) (Sigma, Cat# G8270, USA). Cells were then seeded into the 24-well tissue culture-treated plates (Corning, Cat# 3524, USA) at a density of 5×10^5^ cells per well in 500 μL medium and allowed to adhere for 1.5h. After incubation, floating cells were removed by replacing the medium, leaving only adherent AMs.

### 2.3 High glucose induced immune memory model of alveolar macrophages *in vitro*

The AMs isolated from patients without diabetes were first cultured for 48h in medium containing either 5 mM or 20 mM glucose. After this initial exposure, the cells were washed and re-plated in fresh medium with 5 mM glucose for an additional 5 days. Subsequently, the AMs were stimulated with paraformaldehyde-inactivated *Klebsiella pneumoniae* (*KP*, 10^6^ CFU/mL). Culture supernatants were collected for cytokine analysis, whereas AMs before secondary stimulation were assessed for phagocytosis and real-time ATP production rate.

### 2.4 Isolation and stimulation of alveolar macrophages from diabetic patients

The AMs were derived from patients with or without diabetes, following the previous method. Based on the physiological glucose concentration in lung lining liquid (usually less than 1 mmol/L) (23), the adherent AMs were cultured for 24 h in medium containing 0.6 mM glucose. AMs were also stimulated with paraformaldehyde-inactivated *KP* (10^6^ CFU/mL). Following the same experimental methods described *in vitro* immune memory model, AMs and their supernatants were assessed for cytokines, phagocytosis and real-time ATP production rate.

### 2.5 Cell viability test

Cell viability was assessed using the Cell Counting Kit−8 (CCK−8) assay (Selleck, Cat# B34304, USA). Primary human AMs were seeded into 96−well plates at a density of 6 × 10^4^ cells/well and cultured under two conditions: control (5 mM glucose) and high glucose (20 mM glucose). Then, the viability of AMs was measured at two time points: the 48h of high-glucose stimulation, and the 5-day resting period before second stimulation with paraformaldehyde-inactivated *KP*. At each point, the culture medium was removed, and 100 μL of fresh medium containing 10 μL of CCK−8 solution was added to each well. The plates were incubated for 1h at 37 °C in a humidified atmosphere containing 5% CO_2_. The absorbance of each well was measured at 450 nm using a microplate reader. A blank control (wells containing only culture medium without cells) was included for background subtraction. Cell viability was calculated using the following formula: Cell viability (%) = (OD days– OD blank)/(OD day0 – OD blank) × 100%.

### 2.6 Measurement of cytokine production

Culture supernatants collected from AMs were assessed for inflammatory cytokine levels using a human premixed multi-analyte kit (R&D Systems, Cat# LXSAHM-02, USA), using the manufacturer’s standard protocols. Cytokine concentrations were then quantified on the Luminex 200 multiplex immunoassay platform (Luminex, Cat# 40-012, USA).

### 2.7 Measurement of phagocytosis

Phagocytic activity of tolerant AMs was evaluated using pHrodo Green *E. coli* BioParticles Conjugates (Thermo Fisher Scientific, Cat# P35366, USA), according to the manufacturer’s protocol. Both fluorescent and bright-field images were captured with the Automatic Cell Imaging Analysis System (Alicelligent, Cat# Falcon S400, China). To quantify phagocytic capacity, the mean fluorescence intensity (MFI) and the percentage of green BioParticles-positive cells were determined using the analysis software integrated with the Automatic Cell Imaging Analysis System (Alicelligent, Cat# Falcon S400, China).

### 2.8 Immunofluorescence microscopy

Immunofluorescence staining was performed to examine the localization of CD206, HLA-DR, and CD169. Adherent AMs were fixed with 4% paraformaldehyde (15min), washed with PBS, and permeabilized with 0.2% Triton X-100 in PBS (10min). After blocking with 10% donkey serum for 1h, cells were incubated overnight at 4 °C with primary antibodies against CD206 (Cell Signaling Technology, Cat# 24595T, USA), HLA-DR (Thermo Fisher Scientific, Cat# 17-9956-41, USA), and CD169 (BD Biosciences, Cat# 565248, USA). Following PBS washes, cells were incubated with fluorescent secondary antibodies and DAPI in the dark for 1.5h at room temperature (RT). Images were captured using a confocal laser scanning microscope (Zeiss LSM980, Germany) and analyzed with HALO Image Analysis Platform (Indica Labs, USA).

### 2.9 Transmission electron microscopy

Adherent AM monolayers were directly cultured on the surface of Aclar Embedding Film (Electron Microscopy Sciences, USA), avoiding bubble formation. Once the cultivation period was completed, the cells were fixed using 2.5% (vol/vol) glutaraldehyde dissolved in 0.1 M phosphate buffer (PB). The specimens were rinsed with PB twice and then with deionized water twice to complete the initial cleaning process. Subsequently, cells were kept in contact with a solution containing 1% OsO_4_ and 1.5% potassium ferricyanide (both wt/vol) for a duration of 2 hours at 4 °C. Following this incubation, the cells were then subjected to two rinses in pure acetone, each lasting 6 minutes. Cells were then washed twice in pure acetone (6 min per step). Subsequently, specimens were infiltrated with graded acetone/Spurr’s resin mixtures (10 g ERL 4221, 25 g NSA, 8 g DER 736 and 0.7% DMAE) at ratios of 3:1, 1:1, and 1:3 (v/v), followed by immersion in pure resin. Afterward, the samples were embedded into new Spurr’s resin and polymerized at 45 °C for 12 hours, then continued curing for an extra 48 hours at 70 °C. Ultrathin sections, measuring 70 nm in thickness, were cut using a microtome (Leica EM UC6, Germany) and stained with both lead citrate and uranyl acetate. Subsequently, images were obtained by a transmission electron microscope (TEM) (Tecnai Spirit, USA) that was equipped with an EMSIS CCD camera (EMSIS GmbH, Germany).

### 2.10 RNA isolation and quantitative real-time PCR

Total RNA was extracted from AMs derived from diabetic and non−diabetic individuals using the RNeasy Mini Kit (Qiagen, Cat# 74104, Germany) following the manufacturer’s protocol. The purified RNA was reverse−transcribed into complementary DNA (cDNA) using the PrimeScript RT reagent Kit (Takara, Cat# RR037A, China). Quantitative PCR was then performed with SYBR Green reagent (Takara, Cat# RR820A, China) on an Applied Biosystems 7300 PLUS Real−Time PCR System (Thermo Fisher Scientific, USA). The relative mRNA expression levels of target genes (*PERP, TP53, ATG9A, TFEB, TNF, and IL−1B*) were determined using the 2^^-^ΔΔCt^ method, with actin serving as the internal control. All primer sequences were synthesized by Sangon Biotech (Shanghai, China) and are listed in **Supplementary Table 2**).

### 2.11 Western blotting

Proteins were extracted using Radio Immunoprecipitation Assay (RIPA) buffer containing protease and phosphatase inhibitors (Thermo Fisher Scientific, Cat# 78440, USA). Equal amounts of protein were separated by SDS-PAGE and transferred onto polyvinylidene fluoride (PVDF) membranes. The membranes were blocked with 5% non−fat milk and then incubated with primary antibodies against ULK1 (Abcam, Cat# ab32445, UK) and BAD (Abcam, Cat# ab177472, UK). GAPDH (Proteintech, Cat # 60004−1−Ig, China) was used as the loading control. After incubation with HRP−conjugated secondary antibodies (Abcam, Cat# ab177472, UK) for 2 h at room temperature. The protein bands were visualized using an ECL detection kit (Thermo Fisher Scientific, USA) and the signals were detected with an integrated chemiluminescence imaging system (OI-X6Touch, China).

### 2.12 Metabolic flux measurements of seahorse XFe96

To conduct metabolic flux analysis, alveolar macrophages (AMs) were evaluated via a Seahorse XFe96 Analyzer (Agilent). Prior to the measurement, the cells were cultured following the previously described protocol, then collected and placed into 96-well plates at a cell density of 3.5 × 10^4^ cells per well. To explore the metabolic characteristics, the mitochondrial respiratory function of the seeded cells was determined by the Seahorse XF Cell Mito Stress Test Kit (Alicelligent, Cat# ALS22042, China), which involved sequential injections of 1.5 μmol/L oligomycin (ATP synthase inhibitor), 2.0 μmol/L uncoupling agent (carbonyl cyanide 4-(trifluoromethoxy) phenylhydrazone, FCCP), and 2.0 μmol/L rotenone/antimycin A (Rot/AA, mitochondrial complex I/III inhibitors). Glycolytic activity was assessed via the Seahorse XF Glycolytic Rate Test Kit (Alicelligent, Cat# ALS22022, China), which incorporates the stepwise addition of 1.0 μmol/L rotenone/antimycin A and 50 mM 2-deoxy-D-glucose (2-DG), a glucose derivative that functions as a competitive hexokinase blocker. Cell numbers were normalized utilizing the Automatic Cell Imaging Analysis System (Alicelligent, Cat# Falcon S400, China). Subsequent data processing was conducted automatically via Agilent Wave software (version 2.6).

### 2.13 Untargeted metabolomics analysis

For untargeted metabolomics analysis, samples of AMs obtained from diabetic and non-diabetic individuals were subjected to a 24-hour cultivation period in a glucose-supplemented medium with a concentration of 0.6 mM before undergoing LC-MS/MS analysis. Subsequently, cell lysates were prepared by extracting with ice-cold methanol for 20 minutes on dry ice, followed by rapid freezing in liquid nitrogen. Prior to the LC-MS/MS analysis, the samples were subjected to vortex mixing and centrifugation at 10,000×g (10 min, 4 °C). The resulting supernatant, which included a quality control sample, was then filtered through centrifugation and subsequently transferred into vials intended for LC-MS analysis. A Vanquish Neo UHPLC system (Thermo Fisher Scientific, USA) coupled to an Orbitrap Exploris 120 mass spectrometer (Thermo Fisher Scientific, USA) was used for LC-MS/MS analysis, with chromatographic separation on a Waters ACQUITY UPLC BEH amide column (2.1 mm × 50 mm, 1.7 μm). The mobile phase consisted of solvent A (25 mM ammonium acetate and ammonium hydroxide in water, pH 9.75) and solvent B (acetonitrile). ESI source conditions were as follows: sheath gas flow rate, 50 Arb; auxiliary gas flow rate, 15 Arb; capillary temperature, 320 °C; collision energy, SNCE 20/30/40; and spray voltage, 3.8 kV (positive mode) or -3.4 kV (negative mode). Metabolites were identified according to Metabolomics Standards Initiative (MSI) confidence levels (24). Data processing was subsequently conducted using R software (25). Metabolites exhibiting Variable importance in projection (VIP) scores > 1 and *P* < 0.05 were identified as significantly altered. To further characterize the metabolic alterations, volcano plots, metabolite enrichment analysis, and metabolic pathway analysis were employed. Untargeted metabolomics analysis using LC-MS in this study was supported by Shanghai Biotree Biomedical Technology Co., Ltd.

### 2.14 RNA-seq

Total RNA was isolated using the RNeasy Mini Kit (Qiagen, Germany) according to the manufacturer’s instructions. RNA concentration and integrity were assessed with a NanoDrop spectrophotometer (Thermo Fisher Scientific, USA) and agarose gel electrophoresis, respectively. For library construction, 2 μg of total RNA was processed with the KC-Digital Stranded mRNA Library Prep Kit for Illumina (Wuhan Seqhealth Co., Ltd, China) following the manufacturer’s protocol. The resulting UID mRNA-seq library was quantified on a Qubit 2.0 fluorometer and quality-checked by agarose gel electrophoresis. Sequencing was performed on an Illumina NovaSeq 6000 platform (Illumina, USA) in PE150 mode. Following sequencing, raw reads underwent preprocessing to produce clean reads, where quality control steps included evaluations of base mass distribution and base balance. PCR duplicates were removed using UID information. Clean reads were then aligned to the human reference genome. Differential expression analysis was conducted using the edgeR package (version 3.12.1) (26), using thresholds of *P* < 0.05 and |log_2_FC| > 1. Functional enrichment analysis, including Gene Ontology (GO) and Kyoto Encyclopedia of Genes and Genomes (KEGG) pathway annotations, was carried out with KOBAS software (version 2.1.1), and statistical significance was defined as *P* < 0.05. RNA-seq and data analysis were conducted by Seqhealth Technology Co., LTD (Wuhan, China).

### 2.15 ATAC-seq

To process each biological sample, 20,000 individual cells were subjected to lysis, and the resulting nuclei were collected via centrifugation at 500×g for a duration of 5 minutes. Following the cell lysis and nuclear pelleting steps, transposition and library preparation were conducted using the TruePrep DNA Library Prep Kit V2 for Illumina (Nanjing Vazyme, China). The resulting libraries were enriched, quantified, and sequenced on an Illumina NovaSeq 6000 platform (Illumina, USA) in PE150 mode. Raw reads were preprocessed with fastp (v0.23.1) to remove low-quality reads and adapter sequences. Clean reads were then aligned to the human reference genome using bowtie2 (v2.2.6) with default settings. Read distribution was assessed with RSeQC (v2.6), and insert sizes were estimated using Picard’s Collect Insert Size Metrics tool (v2.8.2). Using DeepTools (v2.4.1), the distribution of reads around transcription start sites (TSS) was visualized. Peak calling was conducted with MACS2 (v2.1.1). Peak annotation and distribution analysis were performed using Bedtools (v2.30.0). Differential peaks were identified with csaw (v1.24.3). Functional enrichment analysis (GO and KEGG) was performed with KOBAS (v2.1.1), with significance set at *P* < 0.05. Once these aligned reads were processed into BAM files, visualization of signal intensity at specific genomic regions became possible. ATAC-seq and data analysis were conducted by Seqhealth Technology Co., LTD (Wuhan, China).

### 2.16 CUT&Tag

For each biological replicate, a quantity of 50,000 individual cells was gathered and subsequently mixed with magnetic microspheres for a 15-minute period at ambient temperature. Following this, the bead-attached cells were resuspended and allowed to incubate overnight at 4 °C with primary antibodies, specifically anti-H3K4me3 (rabbit monoclonal, Cat# 9751, CST) and control IgG (rabbit monoclonal, Cat# 3900S, CST). Subsequently, the unbound primary antibodies were separated using a magnetic separator the following day, after which a secondary antibody (goat anti-rabbit IgG, Abclonal) was introduced and allowed to react (30min, RT). Subsequently, the unbound antibodies were separated by using a magnetic separator. Subsequently, the pA-Tn5 adapter complex was added and allowed to react (1h, RT), following which unbound pA-Tn5 components were eliminated through washing steps. Subsequent to the initial DNA extraction steps, a tagmentation process was carried out by resuspending the isolated cells in a specialized tagmentation buffer and maintaining the mixture at 37 °C for a duration of 1 hour. Following this incubation period, the immunoprecipitated DNA was collected for further processing. To prepare the sequencing library, the recovered DNA was then subjected to amplification. This process involved combining 21 microliters of the extracted DNA with 2 microliters of a universal i5 primer, 2 microliters of a sample-specific barcoded i7 primer, and 25 microliters of PCR Master Mix (2×). Following the preparation of the reaction mixture, thermal cycling was performed using a preheated thermocycler to amplify the DNA fragments. Following library preparation, sequencing was conducted on the Illumina NovaSeq 6000 (Illumina, USA) in PE 150. Following library sequencing, low-quality reads were first processed with Trimmomatic (v0.36) to filter out substandard sequences. Subsequently, the cleaned reads were aligned to the human genome using Bowtie2, and any PCR-generated duplicates were eliminated using SAMtools (v1.3.1). Using these aligned sequences, peak calling was performed with MACS2 (v2.1.2) under standard parameters: bandwidth set at 300 bp, model fold ranges from 5 to 50, and a significance threshold of 1 × 10^-5^. Finally, the identified peaks were annotated by assigning them to the nearest gene based on the proximity of their summits to the Transcription Start Site (TSS). Functional enrichment analysis (GO and KEGG) was performed with the clusterProfiler R package, with statistical significance assessed by hypergeometric test (*P* < 0.05). The BAM files were employed for visualizing signal intensity at defined genomic regions. CUT&Tag and data analysis were conducted by IGENEBOOK Biotechnology Co., Ltd. (Wuhan, China).

### 2.17 Statistical analysis

All statistical analyses were conducted using SPSS Statistics (version 25.0) and GraphPad Prism (version 10.0.1), with results expressed as mean ± standard deviation (SD). When making comparisons between two separate groups, either an unpaired Mann-Whitney U test or a paired Student’s t-test was employed. When analyzing data categorized into distinct groups, the statistical significance of differences was determined using either ordinary two-way ANOVA or two-way repeated measures ANOVA. A two-sided α level of 0.05 was applied for all significance tests, and p*-*value < 0.05 was considered statistically significant. Each of these experiments utilized separate, non-overlapping samples to ensure the validity of the findings. Further details regarding the statistical procedures employed were included within the captions of the figures. For multi-omics datasets (RNA-seq, ATAC-seq, metabolomics, Seahorse) and associated functional assays, the sample size (n=3– biological replicates), except for CUT&Tag (n=2 biological replicates), is explicitly indicated in each figure legend. Therefore, given the exploratory nature of these analyses, the results should be interpreted as hypothesis-generating.

## 3 Results

### 3.1 The isolation and characterization of primary human alveolar macrophages

**Figure 1:**
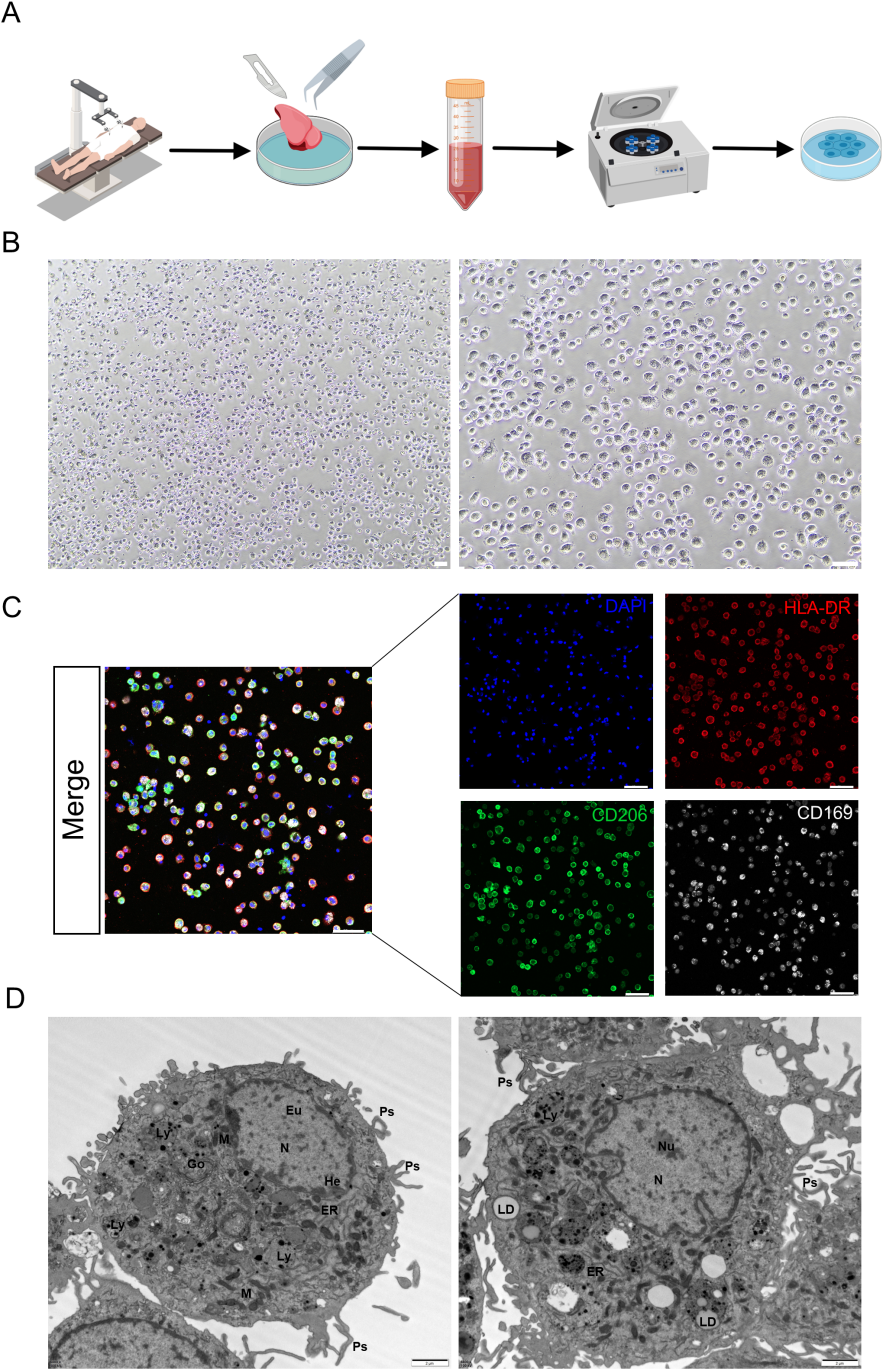
Isolation, culture, and characterization of AMs. **(A)** Graphical overview of the workflow. **(B)** Representative image of adherence. Scale bars, 50 μm. **(C)** Representative image of immunofluorescent microscopy on the normal human AMs. Green is CD206, red is HLA-DR, white is CD169, and blue is nucleus. Scale bar, 50 μm. **(D)** Representative image of transmission electron microscopy of different AMs on the same section. N, nucleus; M, mitochondria; ER, endoplasmic reticulum; LD, lipid droplet; Ly, lysosome; Go, Golgi apparatus; Ps, pseudopodium; He, Heterochromatin; Eu, Euchromatin; Nu, Nucleolus. Scale bar, 2 μm. All experiments were performed with 3 biological replicates.

Bronchoalveolar lavage fluid (BALF) is the conventional method for obtaining primary human AMs. However, BAL is an invasive procedure which is not routinely feasible for patients without corresponding clinical indications. In this study, we utilized lung lobectomy specimens from thoracic surgery to obtain AMs through lung tissue wash fluid, without compromising the requirements of pathological diagnosis. This approach does not increase additional trauma or risk to patients, and the number of cells obtained is sufficient for subsequent experimental requirements, as summarized in **Figure 1A**.

Representative images of lung tissue and the collected washing fluid are shown in **Supplementary Figure 1**. Light microscopy revealed that the cells were round or oval, extended pseudopodia after adherence, exhibited good light transmission, and had heterogeneous cytoplasm, consistent with the morphological characteristics of primary AMs (**Figure 1B**). Subsequently, we maintained the cells *in vitro* using culture media with different glucose concentrations. In culture medium containing 5 mM glucose, AMs were mostly round or oval and not fully spread at 1.5 h. At day 1, they began to spread but remained predominantly round or oval. At day 3, most cells exhibited typical macrophage morphology with abundant cytoplasm and pseudopodia. By day 7, the cells were fully spread and morphologically diverse, including round and elongated spindle-shaped cells. Throughout culture, cells adhered well and had highly refractive cytoplasm (**Supplementary Figure 2A**). And in complete medium containing 0.6 mM glucose, AMs adhered well from 1.5 h to day 1, maintaining a round or oval morphology with highly refractive cytoplasm. No obvious floating cells were observed (**Supplementary Figure 2B**).

Immunofluorescence staining showed that the isolated cells highly expressed the key markers (CD206, HLA-DR, and CD169) (**Figure 1C**), with a triple-positive rate not less than 88%. This result indicated that we obtained a relatively purified population of primary human AMs. TEM further revealed typical pseudopods on the cell surface, nucleus and intracellular organelles including mitochondria, lysosomes, lipid droplets, Golgi body and endoplasmic reticulum (**Figure 1D**).

### 3.2 Hyperglycemia induces innate immune tolerance in human alveolar macrophages

Macrophages orchestrate immune responses through their secretory functions, which shape the local microenvironment and direct the activities of other immune cells (27). Phagocytosis is another essential function of macrophages, serving not only as a primary mechanism for clearing pathogens and apoptotic cells but also as a critical prerequisite for initiating adaptive immune responses (27). To investigate whether hyperglycemia induces immune memory in AMs, we assessed both cytokine secretion and phagocytic capacity in two complementary settings: an *in vitro* high-glucose exposure model and primary AMs isolated from patients with diabetes.

**Figure 2:**
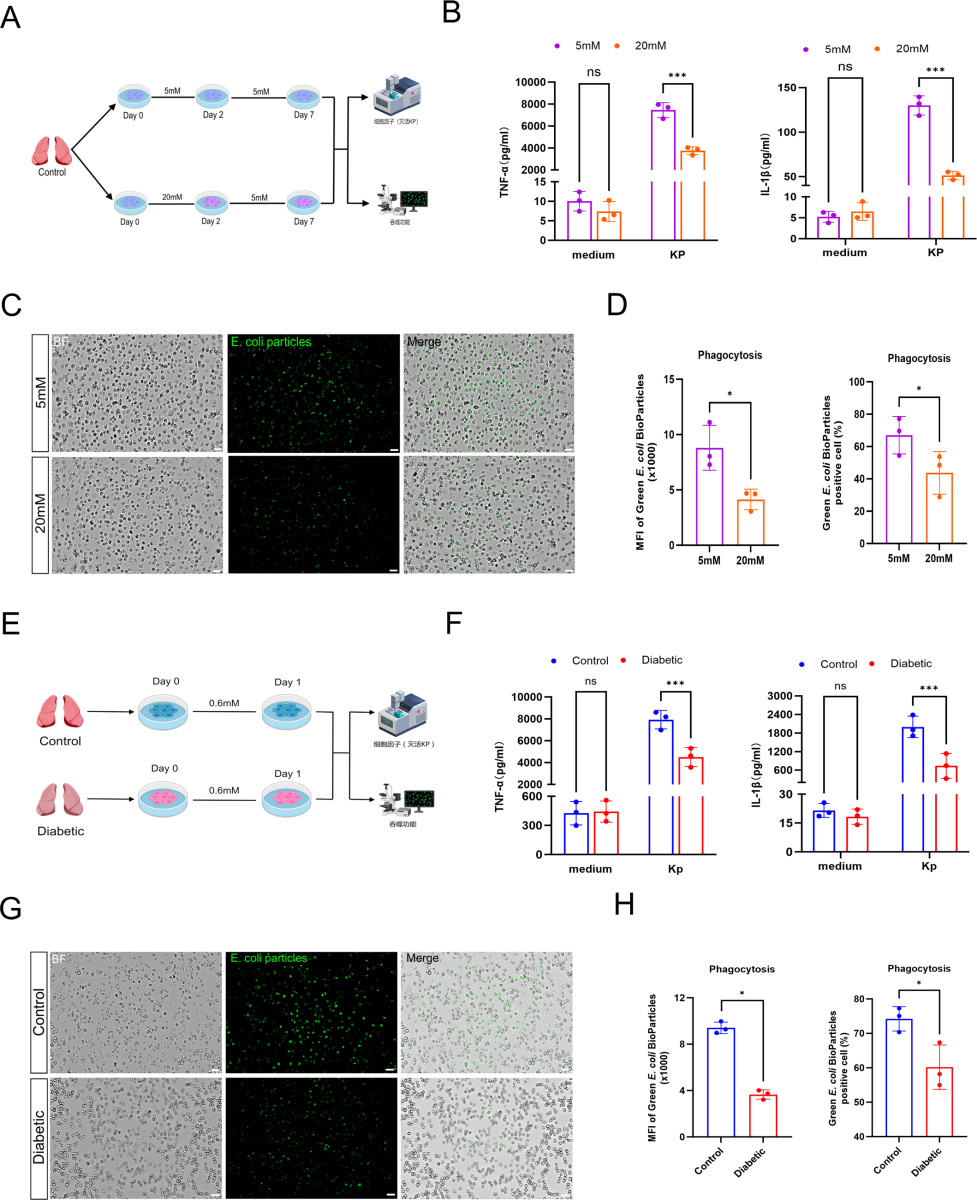
Hyperglycemia alters cytokine secretion and phagocytosis in AMs upon secondary stimulation. **(A)** Graphical overview of the workflow *in vitro* model. **(B)** TNF-α and IL-1β secretion upon secondary stimulation. **(C)** The representative images of AMs were observed under a Falcon S400 Automatic Cell Imaging Analysis System. Scale bar, 50 μm. **(D)** Phagocytic capacity measured by MFI and the percentage of pHrodo-positive cells. P-value in **(B)** was calculated by two-way repeated measures ANOVA, and p-value in **(D)** was calculated by paired Student’s t-test (n = 3 biological replicates for paired samples). **(E)** Graphical overview of the workflow in diabetic. **(F)** The changes of cytokine levels in TNF-α and IL-1β in response to secondary stimulation. **(G)** The representative images of AMs were observed under a Falcon S400 Automatic Cell Imaging Analysis System. Scale bar, 50 μm. **(H)** The phagocytic function was evaluated by MFI and the percentage of green **(E)** coli BioParticles positive cells. P-value in **(F)** was calculated by ordinary two-way ANOVA, and p-value in **(H)** was calculated by Mann-Whitney U test (n = 3 biological replicates). All data are expressed as mean ± SD, ^*^P < 0.05, ^**^P<0.01, ^***^P<0.001, ns, not significant.

Primary AMs were pre-stimulated in medium containing 5 mM or 20 mM glucose for 48 h, followed by a 5-day resting period in 5 mM glucose medium. On day 6, cells were re-stimulated with paraformaldehyde-inactivated KP for 24 h (**Figure 2A**). AMs pre-exposed to 20 mM glucose secreted significantly less TNF-α and IL-1β upon re-stimulation than those cultured in 5 mM glucose (**Figure 2B**). Phagocytic function was assessed by measuring the mean fluorescence intensity (MFI) and the percentage of cells positive for phagocytosed pHrodo Green *E. coli* BioParticles. Consistent with the cytokine profile, AMs pre-exposed to high glucose exhibited a marked reduction in phagocytic capacity (**Figures 2C, D**). Importantly, CCK−8 assays revealed that AMs did not exhibit significant inhibition of cell viability after high−glucose stimulation or before the secondary KP challenge (**Supplementary Figure 3**).

To determine whether this phenotype of immune memory exists *in vivo*, AMs were directly isolated from patients with or without diabetes. Following isolation, cells were rested for 24 h and then subjected to the same KP re-stimulation protocol (**Figure 2E**). AMs from diabetic patients produced significantly lower levels of TNF-α and IL-1β compared with those from the non-diabetic control group (**Figure 2F**). Furthermore, phagocytic activity was also significantly impaired in AMs from diabetic patients, as evidenced by reduced MFI and a lower percentage of phagocytic cells (**Figures 2G, H**). Taken together, these results demonstrate that hyperglycemia induces innate immune tolerance in human AMs, characterized by impaired cytokine secretion and diminished phagocytic function, with consistent findings observed in both the *in vitro* model and cells derived from diabetic patients. In this study, “immune tolerance” is operationally characterized by reduced pro-inflammatory cytokine (TNF-α and IL-1β) secretion and impaired phagocytosis upon secondary stimulation; other immune functions such as ROS production, bactericidal activity, or antigen presentation were not assessed.

### 3.3 Hyperglycemia alters cellular metabolism involved in tolerant alveolar macrophages

**Figure 3:**
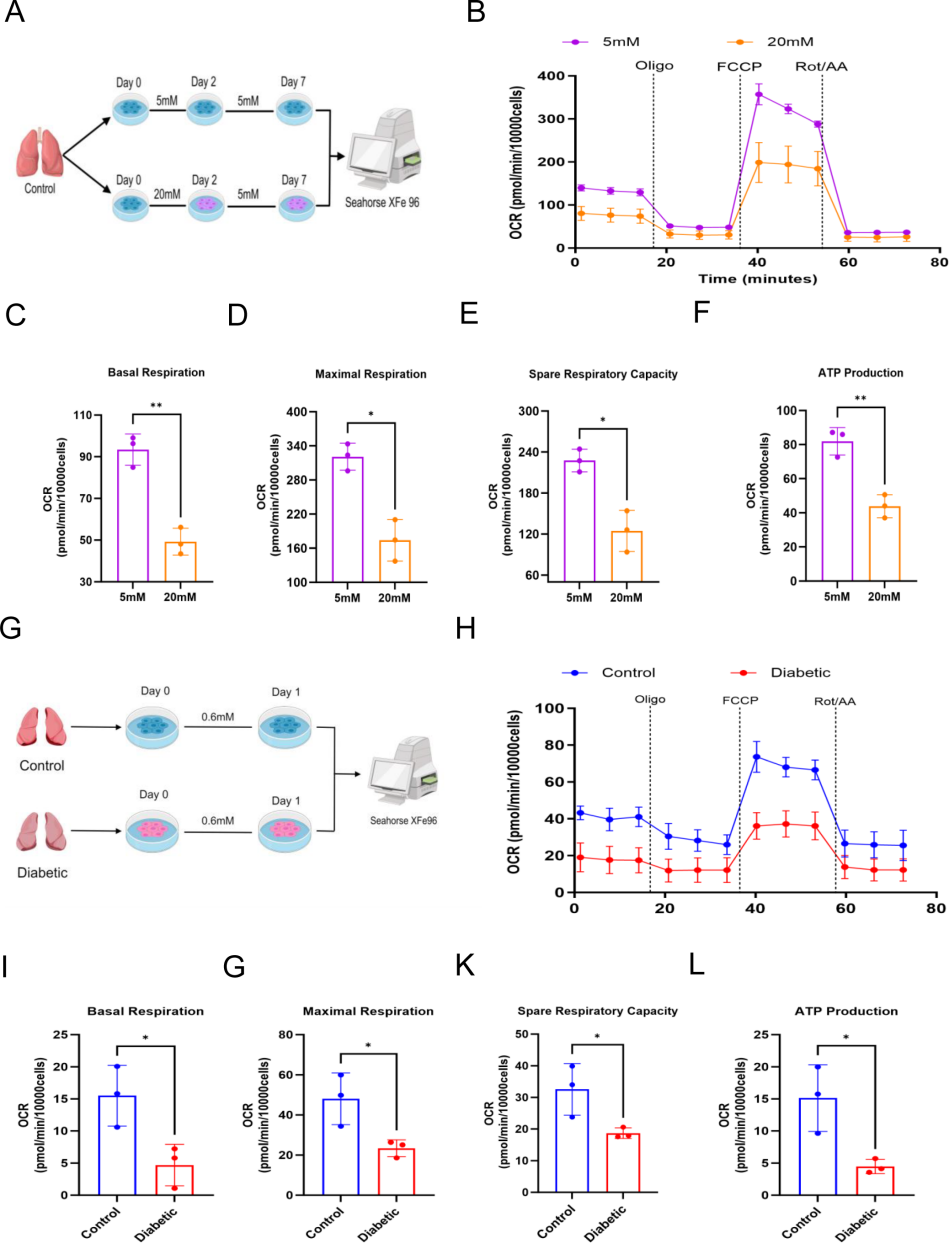
Hyperglycemia regulates the energy metabolism of AMs. **(A)** Graphical overview of the workflow *in vitro* model. **(B–F)** Seahorse energy metabolism was used to assess the impact of high glucose on mitochondrial respiration *in vitro* memory model. P-value was calculated by paired Student’s t-test (n = 3 biological replicates for paired samples). **(G)** Graphical overview of the workflow in diabetic. **(H–L)** Seahorse energy metabolism was used to assess the impact of hyperglycemia on mitochondrial respiration in patients. P-value was calculated by Mann-Whitney U test (n = 3 biological replicates). All data are expressed as mean ± SD, ^*^P<0.05, ^**^P<0.01, ns, not significant.

Innate immune memory in macrophages is maintained through epigenetic modifications, which influence the accessibility of relevant genes to promote or inhibit the transcription of specific genes in response to secondary challenges. Metabolic rewiring is often intertwined with alterations in the epigenetic landscape (28). AMs, as important tissue-resident macrophages in pulmonary innate immunity, have been reported to rely on fatty acid oxidation and OXPHOS for functional maintenance, with limited glycolytic capacity (29, 30). Therefore, we hypothesized that extracellular hyperglycemia may contribute to the transition of AMs to a tolerant phenotype, potentially through alterations in oxidative phosphorylation. To assess OXPHOS and glycolysis, we measured both the oxygen consumption rate (OCR) and the glycolytic proton efflux rate (glycoPER) in live cells. GlycoPER, which is derived by subtracting the mitochondrial proton efflux rate from the total proton efflux rate (31), provides a more accurate reflection of glycolytic activity than the extracellular acidification rate. AMs isolated and enriched by adherence from non-diabetic patients were cultured in medium containing 5 or 20 mmol/L glucose for 2 days. The AMs were then replated into complete medium containing 5 mmol/L glucose for 5 days (**Figure 3A**). In AMs exposed to 20 mM glucose, basal respiration, maximal respiration, spare respiratory capacity, and ATP production were significantly lower than those in the 5 mmol/L group, while basal glycolysis and compensatory glycolysis remained unchanged (**Figures 3B–F**; **Supplementary Figure 4A**). Next, we obtained AMs from patients with or without diabetes using the same methods (**Figure 3G**). These AMs were rested for 24 h in medium containing 0.6 mM glucose. These results were consistent with the high glucose-induced immune tolerance model of AMs *in vitro* (**Figures 3H–L**; **Supplementary Figure 4B**). These results indicated that hyperglycemia-induced immune tolerance in AMs is associated with the decline in OXPHOS.

**Figure 4:**
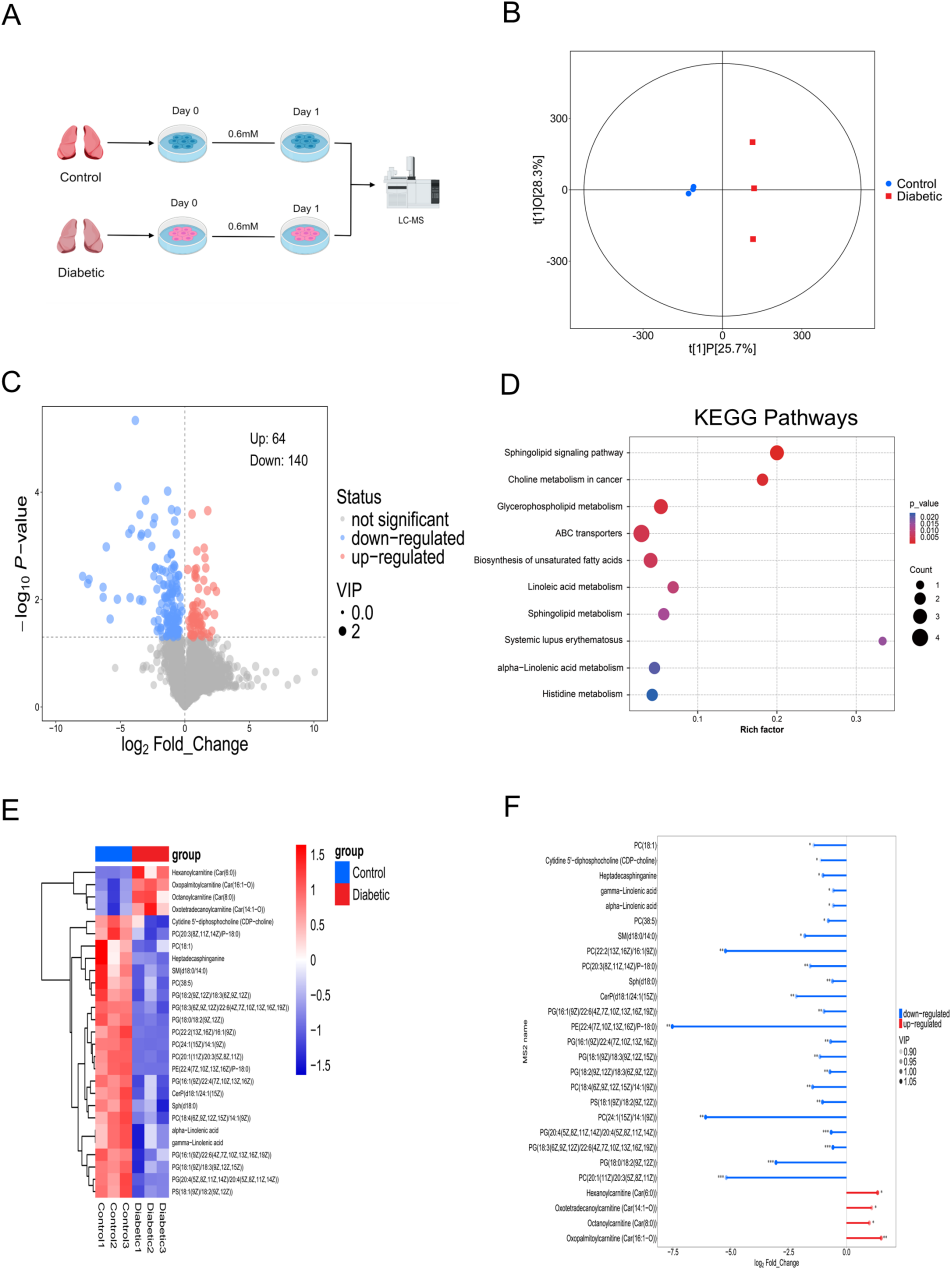
Hyperglycemia alters metabolic profile of AMs. **(A)** LC-MS workflow. **(B)** OPLS-DA score plot meter (R²Y = 0.997, Q² = 0.708; 200-permutation test, *P* < 0.05) confirmed robust predictive ability. The score plot depicts the first predictive component (t [1]P: 25.7%) capturing inter-group variation versus the first orthogonal component (t [1]O: 28.3%) capturing intra-group variation. **(C)** Differentially abundant metabolites volcano plot between the two groups. The significance thresholds were set at p-value < 0.05 (horizontal dashed lines) and VIP > 1. **(D)** The bubble chart of KEGG pathway. **(E)** Heatmap of key metabolites. **(F)** Stem plot of upregulated/downregulated key metabolites. ^*^P < 0.05, ^**^P < 0.01, and ^***^P < 0.001. Data from n=3 biological replicates per group.

To gain deeper insight into the metabolic reprogramming associated with this phenotype, AMs from diabetic and control groups rested for 24 h in medium containing 0.6 mM glucose were subjected to untargeted metabolomics analysis (**Figure 4A**). Based on the metabolomics data, orthogonal partial least squares discriminant analysis (OPLS-DA) was performed, and the resulting score plots demonstrated a distinct separation between the two groups, suggesting substantial variations in their metabolic profiles (**Figure 4B**). An analysis identified a total of 204 differentially changed metabolites (DCMs) with varying abundances across all specimens, consisting of 64 that showed increased levels and 140 displaying decreased expression. These variations were effectively presented through volcano plots and heatmaps between the two groups (**Figure 4C**; **Supplementary Figure 5**). KEGG pathway analysis demonstrated that the identified differential metabolites were significantly enriched in glycerophospholipid metabolism, polyunsaturated fatty acid metabolism, sphingolipid metabolism, and sphingolipid signaling pathway (**Figure 4D**). An analysis of differential abundance (DA) scores further demonstrated that specific metabolites within the mentioned metabolic pathways showed decreased levels (**Supplementary Figure 6**). In detail, we further visualized key metabolites associated with metabolic remodeling in hyperglycemia-induced tolerant AMs using heatmap and stem plot (**Figures 4E, F**; **Supplementary Table 3**). Acylcarnitines were significantly upregulated. Conversely, we observed significantly reduced levels of glycerophospholipids, sphingolipids, lipid metabolism intermediates and polyunsaturated fatty acids.

### 3.4 Transcriptome characteristics of tolerant alveolar macrophages induced by hyperglycemia

**Figure 5:**
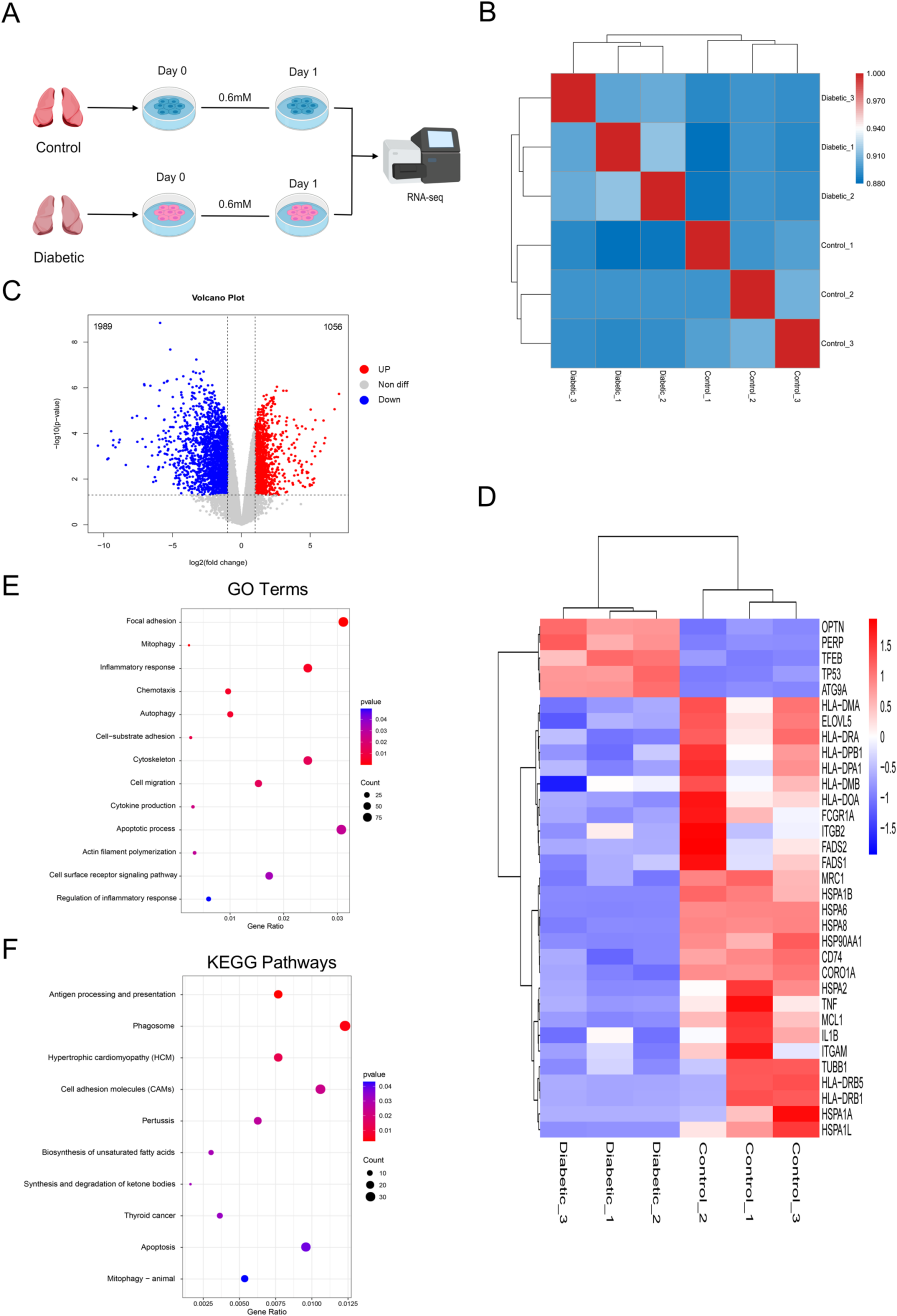
Hyperglycemia alters the transcriptome of AMs. **(A)** RNA-seq workflow. **(B)** Sample correlation heatmap; red indicates higher correlation. **(C)** Volcano plot of DEGs (red: up, blue: down, gray: not significant). **(D)** Heatmap of DEGs; red/blue indicate up/downregulation. **(E)** GO enrichment bubble chart. **(F)** KEGG pathway enrichment bubble chart. **(E, F)** Bubble size represents gene count, color indicates significance (*P* < 0.05). Data from three biological replicates per group.

In order to compare the difference between diabetic patients and non-diabetic patients, we performed RNA sequencing at the same time point as the metabolomics analysis (**Figure 5A**). Heatmap clustering of Pearson correlation coefficients revealed clear separation between the diabetic and control groups, with good within-group sample reproducibility (**Figure 5B**), suggesting that the RNA-seq data are reliable. The distribution of gene expression levels, reflected in the RPKM box plots, also indicated the correlation of the samples and the degree of data dispersion (**Supplementary Figure 7**). A total of 3,045 differentially expressed genes (DEGs) were identified between patients with diabetes and those without diabetes, including 1,989 downregulated genes and 1,056 upregulated genes, as shown in the volcano plot and scatter plot (**Figure 5C**; **Supplementary Figure 8**). In diabetic patients, AMs showed significant upregulation of autophagy- and apoptosis-related genes, whereas AMs from non-diabetic controls upregulated genes involved in inflammation, antigen presentation, phagocytosis, and lipid metabolism. Hierarchical clustering heatmap visualized the expression patterns of these DEGs between two groups (**Figure 5D**). Concurrently, mRNA quantification of several genes indicated that, compared with the control group, tolerant AMs from diabetic exhibited upregulated levels of autophagy- and apoptosis-related genes (e.g., *ATG9A, TFEB, PERP, TP53*), whereas the expression levels of inflammatory genes (e.g., *TNF, IL1B*) were downregulated (**Supplementary Figure 9**). Next, the GO and KEGG pathways were analyzed. GO analysis revealed that DEGs were significantly enriched in multiple biological processes, including phagocytosis, cell motility, autophagy and apoptosis, and immune response (**Figure 5E**). KEGG pathway enrichment analysis showed that DEGs were significantly enriched in pathways related to autophagy and apoptosis, immune response, phagocytosis, lipid metabolism, and cell motility (**Figure 5F**). These GO and KEGG pathway analyses, together with the corresponding DEGs, indicate that hyperglycemia induces systematic transcriptional remodeling in tolerant AMs, providing a transcriptional basis to response to secondary challenges.

### 3.5 Global chromatin shifts of hyperglycemia-induced tolerance in alveolar macrophages

**Figure 6:**
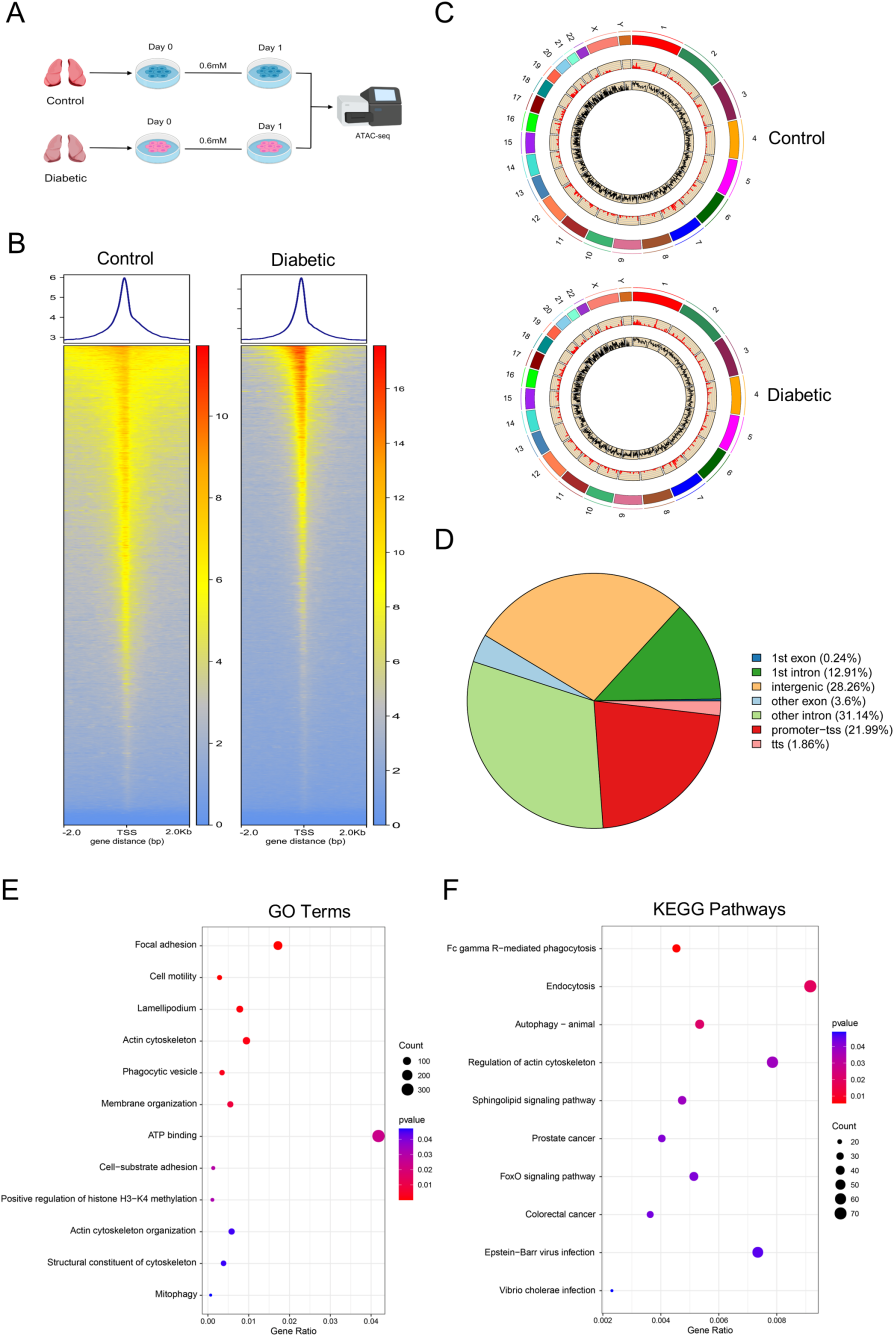
Hyperglycemia alters chromatin accessibility of AMs. **(A)** ATAC-seq workflow. **(B)** Enrichment around TSS and comparison of average signal intensity (X-axis: distance from the TSS, while Y-axis: average signal intensity. **(C)** Distribution of peaks across chromosomes showing chromosome length, peak count, and peak enrichment fold per chromosome (outer to inner). **(D)** Functional annotation of differential peaks. **(E)** Bubble chart showing GO enrichment terms for genes associated with differential peaks. **(F)** Bubble chart depicting KEGG pathway enrichment for the same gene set. **(E, F)** Significance: P < 0.05; bubble size: gene count; color: significance level color. Data from three biological replicates per group.

The openness or condensation state of chromatin structure determines its accessibility, a property that affects how regulatory elements are accessed by transcription factors and other regulatory proteins. To explore if high blood glucose levels correlate with chromatin changes, an investigation using ATAC-seq was conducted on AMs isolated from control and diabetic subjects, with these cells maintained for 24 h (**Figure 6A**). Specifically, the analysis revealed distinct enrichment peaks near the TSS within the -2.0 kb to +2.0 kb interval, with the control group showing a significantly lower average enrichment intensity at the TSS compared to the diabetic group (**Figure 6B**). To explore the modifications in chromatin openness caused by high glucose in AMs that had developed tolerance, an analysis of the identified peaks was conducted to compare their differences. A total of 37,349 and 37,941 accessible chromatin regions (or sites) were identified in the control and diabetic groups respectively, with the distribution of these regions across chromosomes illustrated in **Figure 6C**. These findings suggested that the overall chromatin accessibility was reduced at the chromosomal level in individuals with diabetes. Through differential analysis, a total of 7,800 distinct peaks were identified, where 4,046 chromatin regions displayed elevated accessibility while 3,754 others demonstrated reduced accessibility. Genomic annotation of these differential peaks revealed that 21.99% of them were located in the promoter regions of genes, 44.05% in introns, 28.26% in intergenic regions, and 3.84% in exons (**Figure 6D**). In addition to the genomic location analysis, functional enrichment analysis was performed to explore the biological significance of these differential peaks. The GO analysis indicated that genes associated with these peaks were mainly enriched in biological processes such as cell movement, phagocytosis, autophagy, histone methylation, ATP binding, and membrane organization (**Figure 6E**). Correspondingly, the KEGG analysis revealed significant enrichments of Fc gamma R-mediated phagocytosis, autophagy, regulation of actin cytoskeleton, sphingolipid signaling pathway and FoxO signaling pathway (**Figure 6F**).

### 3.6 Histone H3K4me3 modification of tolerized alveolar macrophages following hyperglycemia exposure

**Figure 7:**
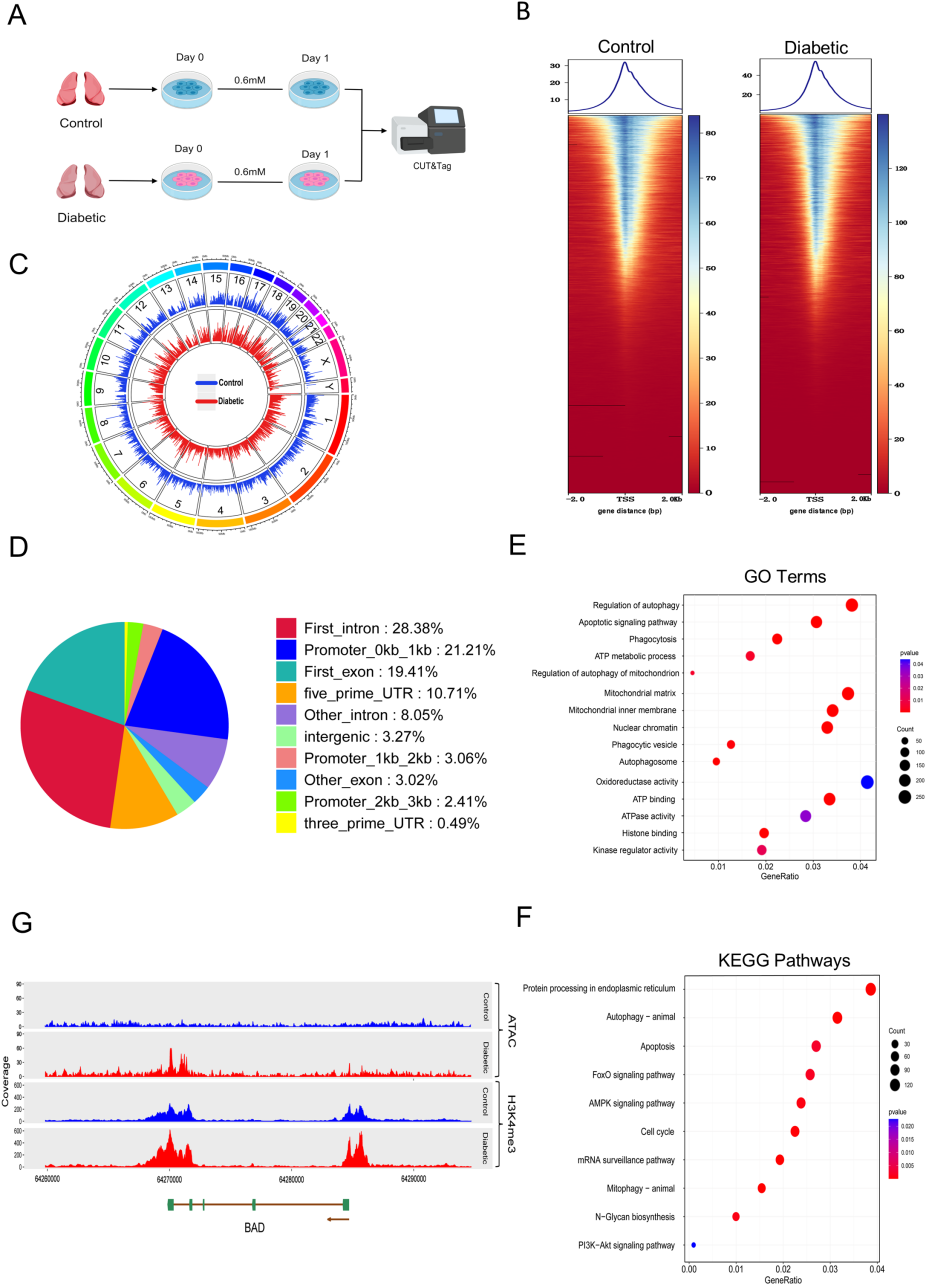
Hyperglycemia alters histone H3K4me3 modification of AMs. **(A)** CUT&Tag workflow. **(B)** Differential H3K4me3 peaks at TSS and comparison of average signal intensity(X-axis: distance from the TSS, while Y-axis: average signal intensity. **(C)** All detected H3K4me3 peaks across chromosomes. Each vertical line represents a peak. **(D)** Distribution and modification of difflerentially enriched H3K4me3 peaks across genomic functional regions. **(E)** Bubble chart showing GO enrichment terms for genes associated with differential peaks. **(F)** Bubble chart depicting KEGG pathway enrichment for the same gene set. **(E, F)** Significance: P < 0.05; bubble size: gene count; color: significance level < color. **(G)** Integrative GenePlots for BAD showing ATAC-seq, H3K4me3 CUT&Tag reads from representative sample. Data from two biological replicates per group.

Furthermore, in order to ascertain the characteristics of hyperglycemia-induced chromatin modification, we analyzed the histone modification (CUT&Tag H3K4me3) in AMs between two groups, consistent with the time point of the previous omics analyses (**Figure 7A**). In control group, the number of peaks of H3K4me3 was 60,037, with a total peak length of 31,469,906 bp and an average length of 524.18 bp. And in diabetic group, the corresponding values were 48,225 peaks, a total length of 28,017,479 bp, and an average length of 580.97 bp. These results indicate that AMs from the control group exhibit more widespread H3K4me3 modifications compared with those from diabetic group overall. In comparison, average peak reads were 343.73 and 412.71 in the control and diabetic groups, respectively, suggesting that AMs have stronger H3K4me3 modification at specific sites in the diabetic group. Consistent with the previous ATAC-seq data, CUT&Tag analysis also revealed distinct enrichment peaks at TSS, with a lower average enrichment level in the control patient group (**Figure 7B**). In order to confirm this finding, we counted the H3K4me3 marks in each chromosome and visualized them (**Figure 7C**). Through differential analysis, 9,506 distinct peaks were identified, of which 9,369 were increased in abundance and 137 were decreased. Subsequent investigation into the genomic annotation of these distinct peaks demonstrated that 26.68% of the peaks were positioned within promoter regions, 36.43% were located in introns, 3.27% were found in intergenic regions, and 22.43% were present in exons (**Figure 7D**). GO enrichment analysis demonstrated that genes linked to the differential peaks between the two groups mainly played roles in processes such as autophagy, programmed cell death, mitochondrial functionality, energy metabolism, phagocytosis, chromatin adjustment, and kinase catalytic activity. Furthermore, KEGG pathway enrichment analysis demonstrated significant enrichment of autophagy, apoptosis, the AMPK signaling pathway, the FoxO signaling pathway, and the PI3K-Akt signaling pathway (**Figures 7E, F**). To further investigate the epigenetic alterations at relevant genomic loci associated with innate immune tolerance, we analyzed the ATAC-seq and H3K4me3 CUT&Tag data at the regulatory regions of ULK1 and BAD implicated in autophagy and apoptosis respectively. Both genes exhibited increased chromatin accessibility accompanied by enriched H3K4me3 modification at their regulatory regions in AMs from diabetic group (**Figure 7G**; **Supplementary Figure 10**). Western blot results (**Supplementary Figure 11**) similarly demonstrated that ULK1 and BAD protein levels were increased in tolerant AMs from diabetic compared with control.

### 3.7 Integrated multi-omics analysis of metabolic changes, transcriptional regulation and epigenetic modification

**Figure 8:**
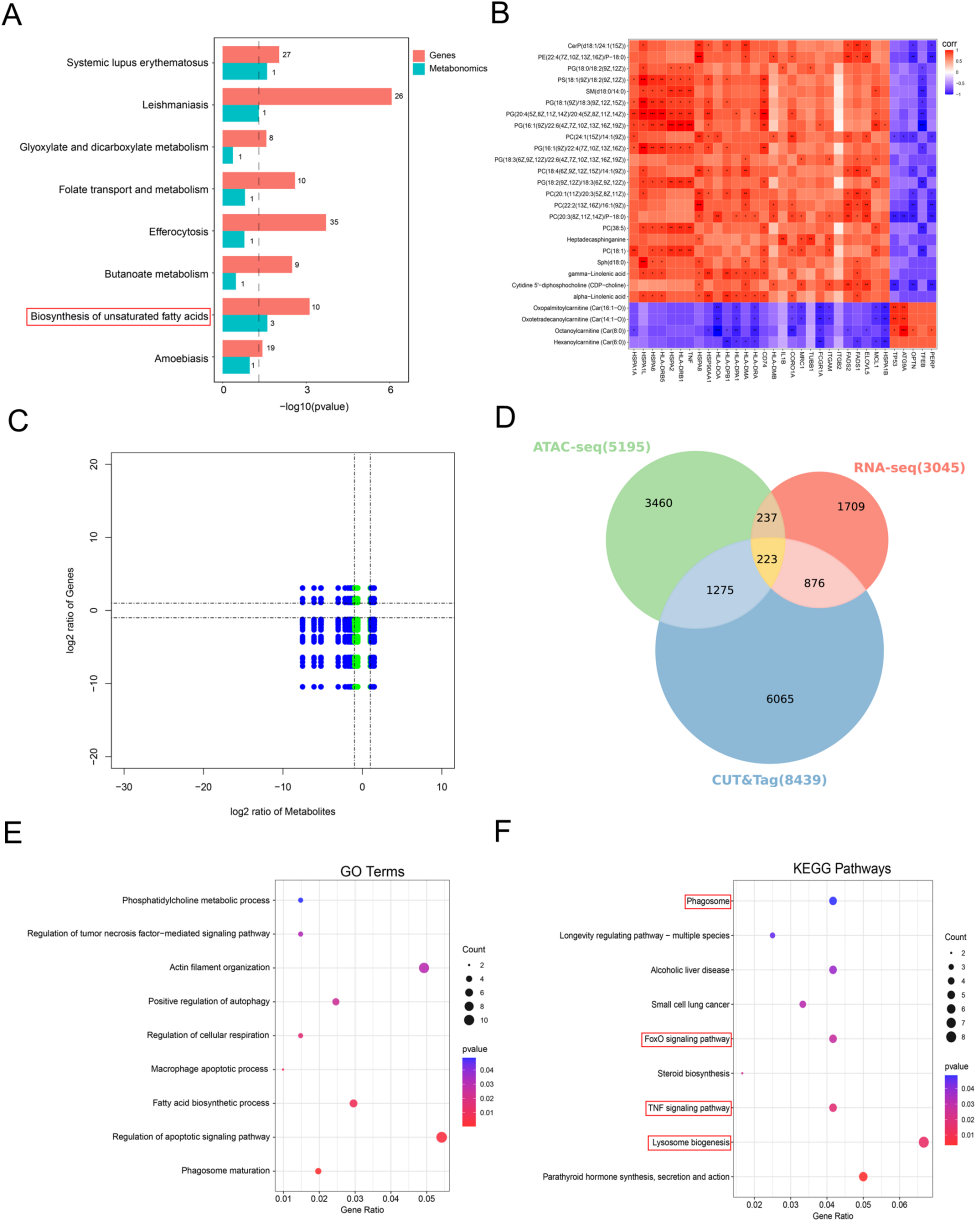
Integrated analysis of multi-omics. **(A)** Integration analysis of DEGs and DCMs KEGG pathways. **(B)** Correlation heatmap of key DEGs and DCMs. **(C)** Nine−quadrant diagram of key DEGs and DCMs (blue: significant in both omics; green: significant in only one). **(D)** Venn diagram showing the overlap among transcriptome, chromatin accessibility, and H3K4me3 modification. **(E)** GO enrichment analysis of differentially expressed genes with different peak-associated genes. **(F)** KEGG analysis of differentially expressed genes with different peak-associated genes. *P < 0.05, **P < 0.01, and ***P < 0.001.

To gain a deeper understanding of the mechanisms underlying hyperglycemia−induced immune tolerance in human AMs, we performed a more comprehensive integrative multi−omics analysis, including integrated analysis of transcriptomics and metabolomics, and combined analysis of transcriptomics and epigenomics. To explore commonly enriched pathways altered between two groups, we used differentially expressed genes and metabolites to map the KEGG database. The results suggested that the biosynthesis of unsaturated fatty acids may regulate the tolerant AMs from diabetic through lipid metabolism (**Figure 8A**). We further performed correlation analysis between the key DEGs in the transcriptome (**Figure 5D**) and the key DCMs in the metabolome (**Figure 4E**). Using Spearman’s correlation analysis, we found that key DEGs related to autophagy and apoptosis were negatively correlated with lipid metabolites (e.g., phosphatidylserine, phosphatidylcholine, phosphatidylglycerol), whereas key DEGs associated with inflammation and immune regulation were positively correlated with these lipid metabolites (**Figure 8B**). The heatmap results suggest that different functional genes may participate in the formation of hyperglycemia-induced tolerant AMs by regulating membrane lipid metabolism. Then, the variations of these DEGs and DCMs in each comparison are illustrated using nine−quadrant diagrams (**Figure 8C**), with |r| >0.8. Results falling into quadrants 3 and 7 indicate a positive association, whereas those in quadrants 1 and 9 indicate a negative association. These results suggest that the biosynthesis of unsaturated fatty acids pathway and key differential genes and metabolites jointly function at the transcriptional and metabolic levels in hyperglycemia-induced tolerant AMs.

Given the synergistic regulation at the transcriptome-metabolome level, we next analyzed the regulatory interactions between the transcriptome and epigenome. We performed Venn chart analysis to identify specific associations among changes in gene expression, changes in chromatin accessibility and H3K4me3 modification in gene regulatory regions. The results revealed a total of 223 overlapping genes, providing a more reliable set of candidate genes for subsequent analysis of the regulatory mechanisms underlying hyperglycemia-induced immune tolerance in AMs (**Figure 8D**). GO enrichment analysis indicated that terms related to autophagy, apoptosis, phagocytosis, cell motility, inflammation, and lipid metabolism may be important in hyperglycemia-induced tolerant AMs (**Figure 8E**). KEGG pathway enrichment analysis further suggested the involvement of phagosome, lysosome, TNF signaling, and FoxO signaling (a known regulator of autophagy and apoptosis) pathways (**Figure 8F**). These findings further suggest that the coordinated regulation between the transcriptome and epigenome may play a role in hyperglycemia-induced tolerant AMs.

## 4 Discussion

Innate immune memory, driven by metabolic and epigenetic reprogramming, is increasingly recognized as a key mechanism in various diseases while program (18, 32). The introduction of the concept “hyperglycemic memory” or “metabolic memory” has been linked to diabetic complications recognize (33). However, compared to circulating mononuclear phagocytes, the specific form of this hyperglycemic memory and its epigenetic basis in tissue-resident AMs of the lung remain unexplored. Here, our results suggested that human AMs also develop a metabolic memory in response to hyperglycemia. This functional remodeling manifests as a tolerant phenotype characterized by reduced levels of inflammatory cytokines and diminished phagocytic capacity, contrasting with the trained immunity reported in other macrophage populations modeling *in vitro*. Our multi-omics data analyze suggest that the mechanisms of tolerance involve selective downregulation of OXPHOS associated with impaired fatty acid beta-oxidation, transcriptional alterations and specific chromatin accessibility patterns which correlate with sustained histone H3K4me3 modifications. Therefore, hyperglycemia-induced immune tolerance of AMs may contribute to the increased risk of pulmonary infections in diabetic patients.

We isolated primary human AMs from lung tissue wash fluid, which better reflect the physiological situation in patients compared to immortalized cell lines, animal models or BMDMs. The approach isolated from wash fluid of tissue specimens serves as a supplementary method for obtaining primary human AMs in specific clinical research scenarios where routine bronchoscopy and bronchoalveolar lavage are not feasible. Functional assays first suggested that hyperglycemia successfully induces an immune tolerant phenotype characterized by decreased inflammatory cytokine secretion and impaired phagocytic function in AMs, in contrast to the trained immunity reported in monocytes and BMDMs (21). Thus, the same stimulus can induce distinct immune memory phenotypes across macrophage lineages. Additionally, acute bacterial infection reprograms alveolar macrophages toward immune paralysis (defective phagocytosis) without altering inflammatory cytokine secretion program towards while (18). These findings reveal that the immune memory phenotype is closely related to both the specific type of stimulus and the intrinsic lineage differences of the cells.

Innate immune memory formation is intimately connected to metabolic reprogramming. A classic example is β−glucan−induced trained immunity, which shifts metabolism from oxidative phosphorylation (OXPHOS) to aerobic glycolysis (the Warburg effect) (34). However, our study identified a distinct metabolic phenotype: a significant decrease in OXPHOS capacity without compensatory glycolysis, both in high glucose-induced AMs and cells from diabetic. This unique phenotype is closely related to the tissue-specific characteristics of AMs, which reside in a high−oxygen, lipid−rich, low−glucose environment and rely heavily on OXPHOS and fatty acid oxidation (FAO) rather than glycolysis (35). Decreased OXPHOS may lead to cellular dysfunction such as reduced cytokine secretion and impaired phagocytosis program. Consistently, cytokine secretion in LPS-stimulated human AMs was also significantly attenuated upon inhibition of OXPHOS (36). The metabolic response of AMs varies with cell origin hemic. For instance, the increased glycolysis was observed in monocyte-derived AMs from influenza-infected mice, as well as in unsorted AMs from bleomycin-treated mice. In contrast, unsorted AMs from saline-treated mice exhibited limited glycolytic capacity (37, 38). These findings showed that metabolic characteristics of tolerant phenotype are shaped by the immune challenge type, tissue environment, and macrophage origin. To further analyze the networks of metabolic remodeling, we performed untargeted metabolomics analysis. Upregulation of various medium- and long-chain acylcarnitines (e.g., Octanoylcarnitine (Car (8:0)), Oxotetradecanoylcarnitine (Car(14:1-O))) was associated with a blockade in the fatty acid β-oxidation, which may limit the availability of substrates for ATP production via OXPHOS and immune function (39, 40). Downregulation of membrane constituents such as glycerophospholipids, sphingolipids, and polyunsaturated fatty acids (41, 42), along with KEGG pathways significantly enriched for lipid metabolism, involves the regulation of membrane fluidity and remodeling, inflammatory signaling, and receptor recognition (43–46), all consistent with the tolerant phenotype we observed. Previous studies have confirmed that increased synthesis of malonyl-CoA inhibited carnitine palmitoyltransferase 1 under high glucose conditions (47, 48), which has been confirmed in pancreatic β-cells and skeletal muscle cells (49, 50). It may represent one of the mechanisms underlying the restricted fatty acid β-oxidation in our study, requiring further clarification. In summary, our metabolic data revealed that hyperglycemia may induce a unique form of metabolic reprogramming, characterized by impaired OXPHOS and lipid metabolic remodeling. These constitute the potential metabolic basis for the suppressed immune function observed in tolerant AMs. It should be noted that we did not directly measure FAO activity (e.g., via radiolabeled palmitate) nor perform pharmacological manipulation of FAO (e.g., CPT1 inhibition/activation) to test its causal role in the tolerant phenotype. Therefore, the involvement of FAO remains correlative.

To explore the gene expression remodeling mechanisms underlying the persistent functional alteration, we performed transcriptomic and epigenetic analyses. RNA-seq analysis revealed significant downregulation of pro-inflammatory cytokine genes (e.g., *TNF, IL1B*), phagocytic receptor genes (e.g., *FCGR1A, ITGB2*), and antigen presentation-related genes (e.g., *HLA-DRA, HLA-DRB1*) in tolerant AMs from diabetic patients, consistent with the observed reduction in cytokine secretion and phagocytosis. This also suggests that the hyperglycemic environment also impairs the antigen-presenting function of macrophages, which is crucial for communication with adaptive immunity. Conversely, autophagy (e.g., *ATG9A, TFEB*) and apoptosis-related (e.g., *PERP, TP53*) genes were significantly upregulated. Enrichment analysis also showed significant enrichment not only in pathways directly related to the tolerant phenotype, such as phagocytosis, antigen processing and presentation, regulation of inflammatory response, cell adhesion, and cell surface receptor signaling pathways, but also in autophagy and apoptosis. Autophagy has been confirmed to maintain cellular homeostasis and regulate immune function (51). Sustained autophagy activation in macrophages can limit inflammatory cytokine production through metabolic reprogramming and post-transcriptional regulation (52) or clearing damaged mitochondria via mitophagy, thereby inhibiting inflammasome activation (53). Similarly, altered apoptotic regulation of macrophage can affect phagocytic function and cytokine expression (54, 55). These studies provide a theoretical basis for understanding the potential involvement of autophagy and apoptosis in maintaining the tolerant phenotype of AMs. Therefore, these findings constitutes the transcriptional basis for the response of tolerant AMs. Epigenomic analyses (ATAC-seq and H3K4me3 CUT&Tag) further analyze revealed significantly enriched terms and pathways, including histone H3-K4 methylation, mitochondrial membrane, energy metabolism, receptor-mediated phagocytosis, cell migration, regulation of mitophagy, apoptosis, FoxO signaling pathway, and AMPK signaling pathway. This suggests that hyperglycemia is associated with systematic alterations in genes related to the immune tolerance phenotype at the levels of chromatin accessibility and H3K4me3 histone modification. Specifically, we identified increased chromatin accessibility and H3K4me3 enrichment at the regulatory regions of BAD (apoptosis) and ULK1 (autophagy initiation) in tolerant AMs. The ULK1 is a key regulatory kinase involved in the initiation of autophagy (56), and BAD participates in apoptosis signal transduction (57). Together, these epigenetic alterations are associated with the initiation of autophagy and apoptosis. To further enhance the reliability of our findings, integrative multi-omics analysis analyze supported these findings (**Figure 8**) analyze signaling. It is important to note that cellular metabolic changes are closely linked to epigenetic regulation. Given that no known direct regulatory relationships exist between the differential metabolites observed in this study (e.g., acylcarnitines, phospholipids) and epigenetic modifying enzymes, the formation of this tolerance mechanism may not involve direct metabolite-epigenetic enzyme interactions. Other potential regulatory mechanisms may include metabolic sensors (e.g., PASK) directly regulating epigenetic modifying enzymes (58), alterations in local nuclear metabolite concentrations directly influencing histone modifications (59), or even that intracellular metabolic and epigenetic remodeling independently regulate cellular responses to secondary challenge (60). These possibilities warrant further investigation.

Although this project provides an initial exploration of the roles of metabolic alterations and epigenetic modifications in hyperglycemia-induced tolerant AMs, several limitations should be acknowledged. The lung tissues were obtained from donors with diverse biological backgrounds (e.g., age, sex, lifestyle, smoking and other environmental exposures). Furthermore, the diabetic and non-diabetic cohorts may differ in variables such as tumor type/stage, comorbidities, and medications (e.g., antidiabetic drugs), which could influence AM phenotype. We did not stratify by these factors due to limited sample size. The term “macroscopically normal lung tissue from lobectomy” is used cautiously to indicate macroscopically tumor-free tissue from lobectomy, not healthy lung. The differences in tissue composition or cell behavior among these donors should also not be ignored behavior among. While the primary human AMs reflect biological characteristics and simulate the unique phenotype and function more accurately, they still cannot fully replicate the complex alveolar microenvironment, such as interactions with lung epithelial cells (61). The establishment and maintenance of immune tolerance memory in AMs require further validation in more complex models, such as alveolar assembloids (62) or organ-on-a-chip systems (63). Additionally, our *in vitro* high-glucose model (20 mM vs. 5 mM glucose) did not include an osmotic control (e.g., mannitol). Thus, we cannot exclude the possibility that some observed effects are partly attributable to hyperosmolar stress rather than glucose-specific signaling. Future studies incorporating osmotic controls are needed to dissect glucose-specific effects. Our characterization of the tolerant phenotype was limited to reduced TNF-α/IL-1β secretion and impaired phagocytosis. And we did not evaluate other relevant functional parameters, including ROS production, bactericidal activity against live bacteria, or antigen presentation capacity (e.g., HLA-DR expression, co-stimulatory molecules, T cell activation). Thus, the term “immune tolerance” in this study is operational and restricted to the measured parameters. Broader functional profiling is required to fully define the tolerant state in human AMs. Finally, the small sample size (n=3) for most multi-omics and functional experiments limits statistical power and generalizability, making findings exploratory. No functional perturbation experiments (e.g., inhibition of fatty acid oxidation or epigenetic enzymes) were performed to establish causality, which is an important direction for our future studies. Key differential metabolites and pathway enrichments (RNA-seq and metabolomics) did not survive multiple testing correction (e.g., FDR), potentially due to small sample size, inter-individual heterogeneity, and moderate hyperglycemic stimulation. Thus, our results may be subject to false positives and require validation in larger cohorts. We expect that the newly identified hyperglycemia-induced tolerance reported in this research will gain increased attention and further elucidation in the future, and that single-cell multi-omics and spatial transcriptomics will help dissect the heterogeneity of immune tolerant macrophages (64, 65).

With the continuous emergence of drug-resistant pathogens, iterative upgrades in anti-infective therapy alone have failed to achieve breakthrough progress (66). Modulating immune status may offer promising avenues for developing anti-infective treatments for diabetic patients with pulmonary infections (67). The feasible therapeutic strategies include: (1) using cell surface receptor agonists or supplementing immunostimulatory factors, (2) recruiting normal immune cells to remodel the local immune microenvironment, (3) employing targeted pharmacological interventions to alter the epigenetic landscape, and (4) modulating metabolic program to appropriately increase inflammatory cytokine expression.

## 5 Conclusions

**Figure 9:**
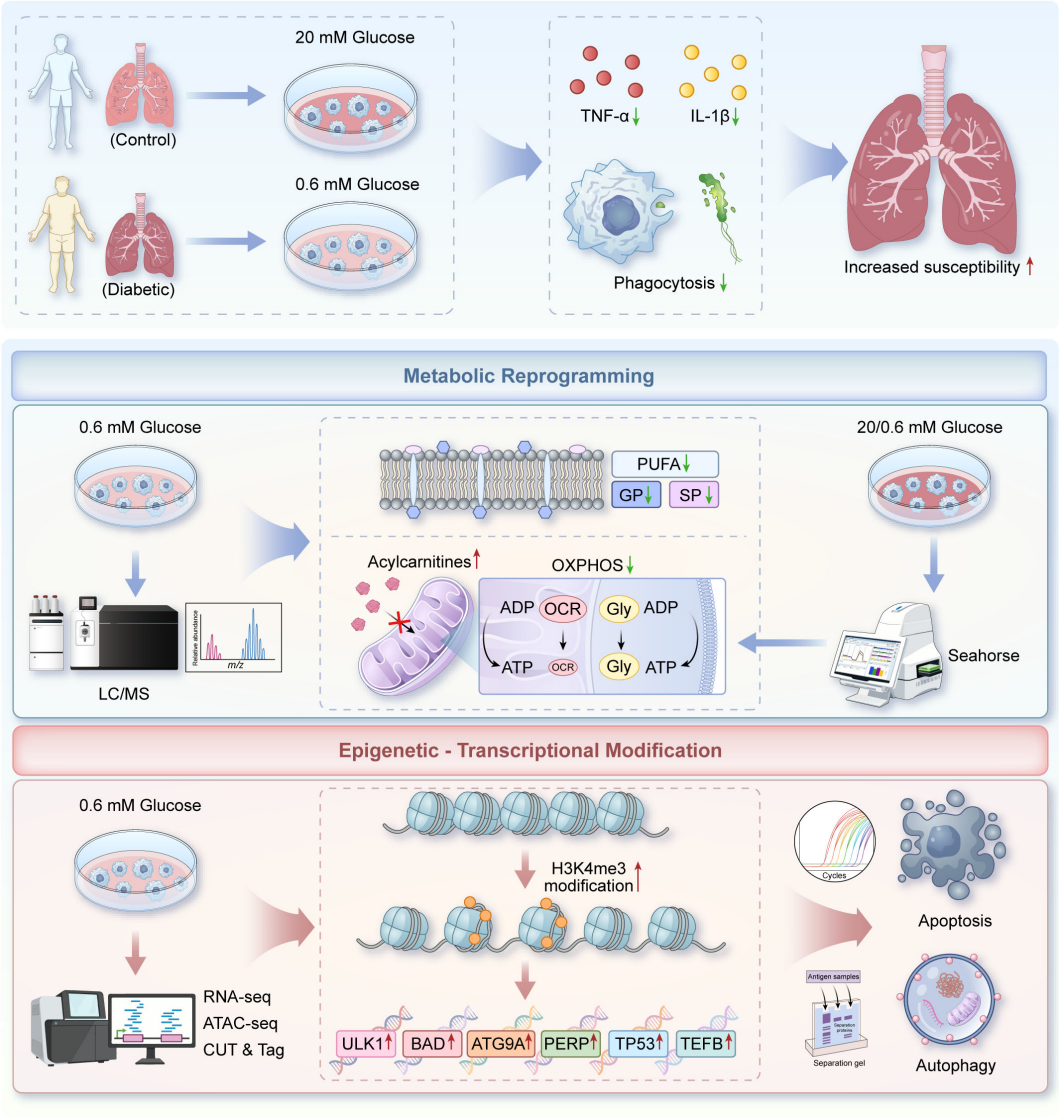
Graphical representation of the regulation mechanism underlying hyperglycemia-induced immunological tolerance in human AMs. Hyperglycemia induces an immune−tolerant phenotype in human AMs, characterized by reduced secretion of pro−inflammatory cytokines (e.g., TNF−α, IL−1β) upon secondary bacterial challenge, along with impaired phagocytosis. Mechanistically, chronic hyperglycemia drives metabolic reprogramming, including reduced OXPHOS with no compensatory increase in glycolysis, as well as lipid metabolism disorders (acylcarnitine accumulation and downregulation of membrane lipids). Additionally, tolerant AMs also exhibit epigenetic reprogramming, manifested as increased chromatin accessibility and enhanced H3K4me3 enrichment at the regulatory regions of autophagy and apoptosis−related genes (e.g., ULK1, BAD). Together, these metabolic and epigenetic alterations are associated with compromised the energy supply and functional capacity of AMs, thereby increasing the susceptibility of diabetic patients to pulmonary infections. OXPHOS, oxidative phosphorylation; AMs, alveolar macrophages; PUFA, polyunsaturated fatty acids; GP, glycerophospholipids; SP, sphingolipids.

In conclusion, our study revealed that hyperglycemia induced immune tolerance in human AMs, characterized by reduced inflammatory cytokine secretion, impaired phagocytosis, and diminished oxidative phosphorylation. Mechanistically, this is associated with program fatty acid oxidation disorders, membrane lipid metabolism abnormalities, and epigenetic modifications of autophagy and apoptosis-related genes (e.g., *ULK1*, *BAD*). These findings reveal a previously unrecognized mechanism underlying AM dysfunction in patients with diabetes, providing a theoretical basis for the increased susceptibility to pulmonary infections and experimental support for the development of prevention and targeted therapy against diabetes−associated pulmonary infections (**Figure 9**).

## Data Availability

The datasets presented in this study can be found in online repositories. The names of the repository/repositories and accession number(s) can be found below: OMIX014762 (https://ngdc.cncb.ac.cn/omix) and HRA016574 (https://ngdc.cncb.ac.cn/gsa-human).

## Supplementary material

Supplementary Figure 1 Representative images of lung tissue from lobectomy and the collected washing fluid (n = 3 biological replicates).

Supplementary Figure 2 Representative images of maintenance culture in complete medium with different glucose concentrations (n = 3 biological replicates). Scale bar, 100 μm.

Supplementary Figure 3 CCK-8 assay was performed to assess AMs viability after 2 days of high glucose (20mM) exposure **(A)** and a subsequent 5 days resting period in normal glucose (5mM) medium **(B)**. P-value was calculated by paired Student’s t-test. All data were presented as mean ± SD (n = 3 biological replicates with paired samples). ns, not significant.

Supplementary Figure 4 GlycoPER measurement by Seahorse XFe96 analyzer. **(A)** Basal and compensatory glycolysis in immune memory model under 5mM vs 20mM glucose (n = 3 biological replicates with paired samples). **(B)** Basal and compensatory glycolysis in patients with or without diabetes (n = 3 biological replicates). All data were presented as mean ± SD. P-values: paired t-test **(A)**, Mann-Whitney U test **(B)**. ns, not significant.

Supplementary Figure 5 Heatmap analysis of the differentially abundant metabolites. The significance thresholds were set at p-value < 0.05 (horizontal dashed lines) and VIP > 1 (n = 3 biological replicates).

Supplementary Figure 6 KEGG pathway DA scoring. The X-axis represents the regulation across the entire pathway (positive values correspond to upregulation; negative values to downregulation). Bar length reflects the magnitude of the DA score. ^*^*P* < 0.05, ^**^*P* < 0.01, ^***^*P* < 0.001 (n = 3 biological replicates).

Supplementary Figure 7 RPKM, Reads per kilobase per million reads values were calculated for boxplot visualization of gene expression distributions across individual samples (X-axis: the sample name, Y-axis: log10 (RPKM). For each box, the center line indicates the median, box boundaries represent the upper and lower quartiles, and whiskers denote the maximum and minimum values (n = 3 biological replicates).

Supplementary Figure 8 Visualizations of gene expression levels are presented through scatter plots of RPKM values. Genes that were significantly downregulated are shown as blue dots, upregulated genes as red dots, and those with no significant expression change as gray dots (n = 3 biological replicates).

Supplementary Figure 9 RT-qPCR analysis of gene expression levels of PERP, TP53, ATG9A, TFEB, TNF and IL1B. P-value was calculated by unpaired Mann-Whitney U test. All data were presented as mean ± SD (n = 3 biological replicates), **P* < 0.05.

Supplementary Figure 10 Integrative GenePlots for ULK1 showing ATAC-seq, H3K4me3 CUT&Tag reads from representative samples of AMs between two groups (n = 3 biological replicates for ATAC-seq and n = 2 biological replicates for CUT&Tag).

Supplementary Figure 11 Western blot analysis of ULK1 and BAD. P-value was calculated by unpaired Mann-Whitney U test. All data were presented as mean ± SD (n = 3 biological replicates), ^*^*P* < 0.05.
