# Supplementary material for: Hyperglycemia- induced innate immune tolerance involves the metabolic and epigenetic rewiring in human alveolar macrophages
Source: Front Immunol. 2026 May 7;17:1834572. doi: 10.3389/fimmu.2026.1834572 (PMC13189875; doi:10.3389/fimmu.2026.1834572)
Supplement: Supplementary file 1 [file Table1.docx]

Table S1 The demographic and clinical characteristics of patients with or without diabetes

|  | Control group  (n=12) | Diabetic group  (n=12) | P value |
| --- | --- | --- | --- |
| Age, (years) | 54.50 ± 6.02 | 55.67 ± 5.63 | 0.6288^#^ |
| Sex, male n (%) | 7 (46.67) | 8 (53.33) | ＞0.99^*^ |
| Height (cm) | 166.7 ±9.10 | 164.5 ±7.61 | 0.5363^#^ |
| Weight (kg) | 67.71 ± 9.02 | 66.54 ± 8.12 | 0.7423^#^ |
| BMI | 24.29 ± 1.94 | 24.58 ± 2.50 | 0.7531^#^ |
| Fasting venous blood glucose (mmol/L) | 4.91 ± 0.44 | 7.18 ± 0.74 | ＜0.0001^#^ |
| HbA1c (%) | 5.18 ± 0.68 | 8.99 ± 1.06 | ＜0.0001^#^ |
| Smoking history n (%) | 6 (50.00) | 5 (41.67) | ＞0.99^*^ |
| Types of DM |  |  |  |
| T1DM | 0 | 0 |  |
| T2DM | 0 | 12 |  |
| Histological type,  n (%) |  |  | ＞0.99^*^ |
| Adenocarcinoma | 9 (75.00) | 10 (83.33) |  |
| Squamous carcinoma | 3 (25.00) | 2 (16.67) |  |
| Others | 0 | 0 |  |
| TNM classification,  n (%) |  |  | ＞0.99^*^ |
| T1N0M0 | 7 | 6 |  |
| T2N0M0 | 5 | 6 |  |
| Antidiabetic drug classes |  |  | N/A |
| Metformin | N/A | 4 |  |
| Alpha-glucosidase inhibitors | N/A | 3 |  |
| Other oral antidiabetic drugs | N/A | 2 |  |
| Insulin | N/A | 2 |  |
| Combination therapy | N/A | 0 |  |
| Untreated | N/A | 1 |  |
| Comorbidities, n (%) |  |  |  |
| Hypertension | 4 (33.33) | 6 (50.00) | 0.6802^#^ |
| CAD | 3 (25.00) | 8 (66.67) | 0.0955^#^ |

^*^Fisher's exact test; ^#^Student's t-test; BMI: Body mass index; T1DM: Type 1 diabetes mellitus; T2DM: Type 2 diabetes mellitus; T: Tumor, N: Node; M: Metastasis; N/A: Not applicable; CAD: Coronary artery disease.
