# Supplementary material for: Hyperglycemia- induced innate immune tolerance involves the metabolic and epigenetic rewiring in human alveolar macrophages
Source: Front Immunol. 2026 May 7;17:1834572. doi: 10.3389/fimmu.2026.1834572 (PMC13189875; doi:10.3389/fimmu.2026.1834572)
Supplement: Supplementary file 2 [file Table2.docx]

| Genes | Sequence（5’ 3’） |
| --- | --- |
| ACTB | F:CGTGGACATCCGCAAAG |
|  | R:AAGGTGGACAGCGAGGC |
| PERP | F:GGAAATGCTCCCAAGAGG |
|  | R:AGGATGATGAAGCCACAGAA |
| TP53 | F:CCACCATCCACTACAACTACAT |
|  | R:AAACACGCACCTCAAAGC |
| ATG9A | F:ATTGGCATCGCTAACTTCC |
|  | R:TCCCGCTTCAGCACCTCA |
| TFEB | F:AATGACCGCATCAAGGAGTT |
|  | R:TGGATACGGAGCCAGAGC |
| TNF | F:CCACCACGCTCTTCTGC |
|  | R:GCTTGAGGGTTTGCTACAAC |
| IL1B | F:ACAGTGGCAATGAGGATG |
|  | R:TGTAGTGGTGGTCGGAGA |

Table S2 Primer sequence
