# Supplementary material for: Hyperglycemia- induced innate immune tolerance involves the metabolic and epigenetic rewiring in human alveolar macrophages
Source: Front Immunol. 2026 May 7;17:1834572. doi: 10.3389/fimmu.2026.1834572 (PMC13189875; doi:10.3389/fimmu.2026.1834572)
Supplement: Supplementary file 3 [file Table3.docx]

Table S3 The quantitative data of key differential metabolites

| MS2 name | Control 1 | Control 2 | Control 3 | Diabetic  1 | Diabetic 2 | Diabetic 3 | VIP | P-Value | Q-Value | Fold_Change |
| --- | --- | --- | --- | --- | --- | --- | --- | --- | --- | --- |
| Hexanoylcarnitine (Car(6:0)) | 2.65E-05 | 2.6E-05 | 2.84E-05 | 8.31733E-05 | 5.07E-05 | 7.15E-05 | 1.881471 | 0.047706 | 0.645016 | 2.53953228 |
| Octanoylcarnitine (Car(8:0)) | 5.22E-06 | 3.39E-06 | 5.14E-06 | 1.00139E-05 | 1.01E-05 | 7.12E-06 | 1.784122 | 0.017336 | 0.459806 | 1.981588914 |
| Oxotetradecanoylcarnitine (Car(14:1-O)) | 5.66E-06 | 3.12E-06 | 5.98E-06 | 9.76919E-06 | 1.28E-05 | 8.83E-06 | 1.733014 | 0.02088 | 0.491287 | 2.126393374 |
| Oxopalmitoylcarnitine (Car(16:1-O)) | 7.15E-06 | 3.92E-06 | 8E-06 | 1.7669E-05 | 1.91E-05 | 1.71E-05 | 1.858067 | 0.001102 | 0.153643 | 2.825684799 |
| alpha-Linolenic acid | 0.00026 | 0.000292 | 0.000302 | 0.000154 | 0.000225 | 0.000196 | 1.718884 | 0.018056 | 0.467363 | 0.673406994 |
| Cytidine 5'-diphosphocholine (CDP-choline) | 1.47E-05 | 1.71E-05 | 1.44E-05 | 1.22E-05 | 4.86E-06 | 4.3E-06 | 1.562658 | 0.037826 | 0.59934 | 0.464015831 |
| gamma-Linolenic acid | 0.00026 | 0.000292 | 0.000302 | 0.000154 | 0.000225 | 0.000196 | 1.718884 | 0.018056 | 0.467363 | 0.673406994 |
| Sph(d18:0) | 0.000117 | 0.000111 | 0.000124 | 8.14E-05 | 8.67E-05 | 6.3E-05 | 1.761343 | 0.007417 | 0.338884 | 0.656692004 |
| PC(18:1) | 1.54E-05 | 8.27E-06 | 9.91E-06 | 3.23E-06 | 2.77E-06 | 6.63E-06 | 1.642992 | 0.047427 | 0.643636 | 0.376703881 |
| Heptadecasphinganine | 7.24E-06 | 4.49E-06 | 5.58E-06 | 3.06E-06 | 2.96E-06 | 2.55E-06 | 1.770141 | 0.023404 | 0.50822 | 0.495050751 |
| PC(38:5) | 1.01E-05 | 7.83E-06 | 8.35E-06 | 4.53E-06 | 6.15E-06 | 4.56E-06 | 1.772281 | 0.013563 | 0.422112 | 0.579546858 |
| PC(20:3(8Z,11Z,14Z)/P-18:0) | 8.77E-05 | 0.000119 | 9.52E-05 | 5.28E-05 | 1.76E-05 | 3.12E-05 | 1.723872 | 0.008728 | 0.356064 | 0.336713599 |
| PC(22:2(13Z,16Z)/16:1(9Z)) | 4.03E-05 | 4.85E-05 | 5.76E-05 | 2.51E-06 | 1.01E-06 | 3.77E-07 | 1.887432 | 0.009875 | 0.36823 | 0.026644971 |
| PC(20:1(11Z)/20:3(5Z,8Z,11Z)) | 3.31E-06 | 3.94E-06 | 3.96E-06 | 9.14E-08 | 2.12E-07 | 3.08E-09 | 1.706785 | 7.94E-05 | 0.070215 | 0.027290991 |
| PG(18:2(9Z,12Z)/18:3(6Z,9Z,12Z)) | 2.27E-05 | 1.97E-05 | 2.03E-05 | 1.13E-05 | 1.46E-05 | 1.23E-05 | 1.852928 | 0.003761 | 0.259382 | 0.609772939 |
| PC(18:4(6Z,9Z,12Z,15Z)/14:1(9Z)) | 5.96E-06 | 7.11E-06 | 7.72E-06 | 2.55E-06 | 3.28E-06 | 1.69E-06 | 1.831675 | 0.00306 | 0.239856 | 0.362356361 |
| PG(18:3(6Z,9Z,12Z)/22:6(4Z,7Z,10Z,13Z,16Z,19Z)) | 2.76E-05 | 2.7E-05 | 2.69E-05 | 1.82E-05 | 1.92E-05 | 1.68E-05 | 1.931785 | 0.000225 | 0.087532 | 0.666090312 |
| PG(16:1(9Z)/22:4(7Z,10Z,13Z,16Z)) | 0.000229 | 0.000218 | 0.00023 | 0.000124 | 0.000169 | 0.000128 | 1.822163 | 0.004353 | 0.27611 | 0.620654326 |
| PC(24:1(15Z)/14:1(9Z)) | 7.01E-05 | 7.03E-05 | 7.77E-05 | 1.57E-06 | 4.67E-07 | 1.17E-06 | 1.940169 | 0.001047 | 0.150553 | 0.014684271 |
| PG(16:1(9Z)/22:6(4Z,7Z,10Z,13Z,16Z,19Z)) | 2.96E-05 | 2.66E-05 | 2.78E-05 | 1.04E-05 | 1.4E-05 | 1.85E-05 | 1.77767 | 0.005451 | 0.30269 | 0.510251031 |
| PG(20:4(5Z,8Z,11Z,14Z)/20:4(5Z,8Z,11Z,14Z)) | 0.000128 | 0.000122 | 0.000137 | 7.73E-05 | 8.34E-05 | 8.4E-05 | 1.928895 | 0.000602 | 0.127121 | 0.632183743 |
| PG(18:1(9Z)/18:3(9Z,12Z,15Z)) | 0.000112 | 0.000108 | 0.000118 | 3.2E-05 | 6.5E-05 | 5.56E-05 | 1.757234 | 0.003808 | 0.259439 | 0.451274016 |
| SM(d18:0/14:0) | 1.88E-06 | 1.1E-06 | 1.5E-06 | 2.75E-07 | 6.07E-07 | 4.06E-07 | 1.793271 | 0.012004 | 0.40201 | 0.287030136 |
| PS(18:1(9Z)/18:2(9Z,12Z)) | 7.14E-05 | 6.43E-05 | 7.68E-05 | 2.89E-05 | 3.82E-05 | 3.64E-05 | 1.892255 | 0.001393 | 0.173632 | 0.487123031 |
| PG(18:0/18:2(9Z,12Z)) | 9.63E-06 | 7.85E-06 | 8.8E-06 | 1.05E-06 | 1.35E-06 | 7.75E-07 | 1.938859 | 0.000141 | 0.086279 | 0.12095677 |
| PE(22:4(7Z,10Z,13Z,16Z)/P-18:0) | 4.82E-05 | 5.88E-05 | 6.11E-05 | 5.48E-07 | 1.98E-07 | 1.65E-07 | 1.948316 | 0.00503 | 0.294626 | 0.005416715 |
| CerP(d18:1/24:1(15Z)) | 3.54E-05 | 3.76E-05 | 4.56E-05 | 4.75E-06 | 1.87E-05 | 2.85E-06 | 1.671656 | 0.006414 | 0.320027 | 0.222148446 |
