# Supplementary figures and images for: Hyperglycemia- induced innate immune tolerance involves the metabolic and epigenetic rewiring in human alveolar macrophages
Source: Front Immunol. 2026 May 7;17:1834572. doi: 10.3389/fimmu.2026.1834572 (PMC13189875; doi:10.3389/fimmu.2026.1834572)

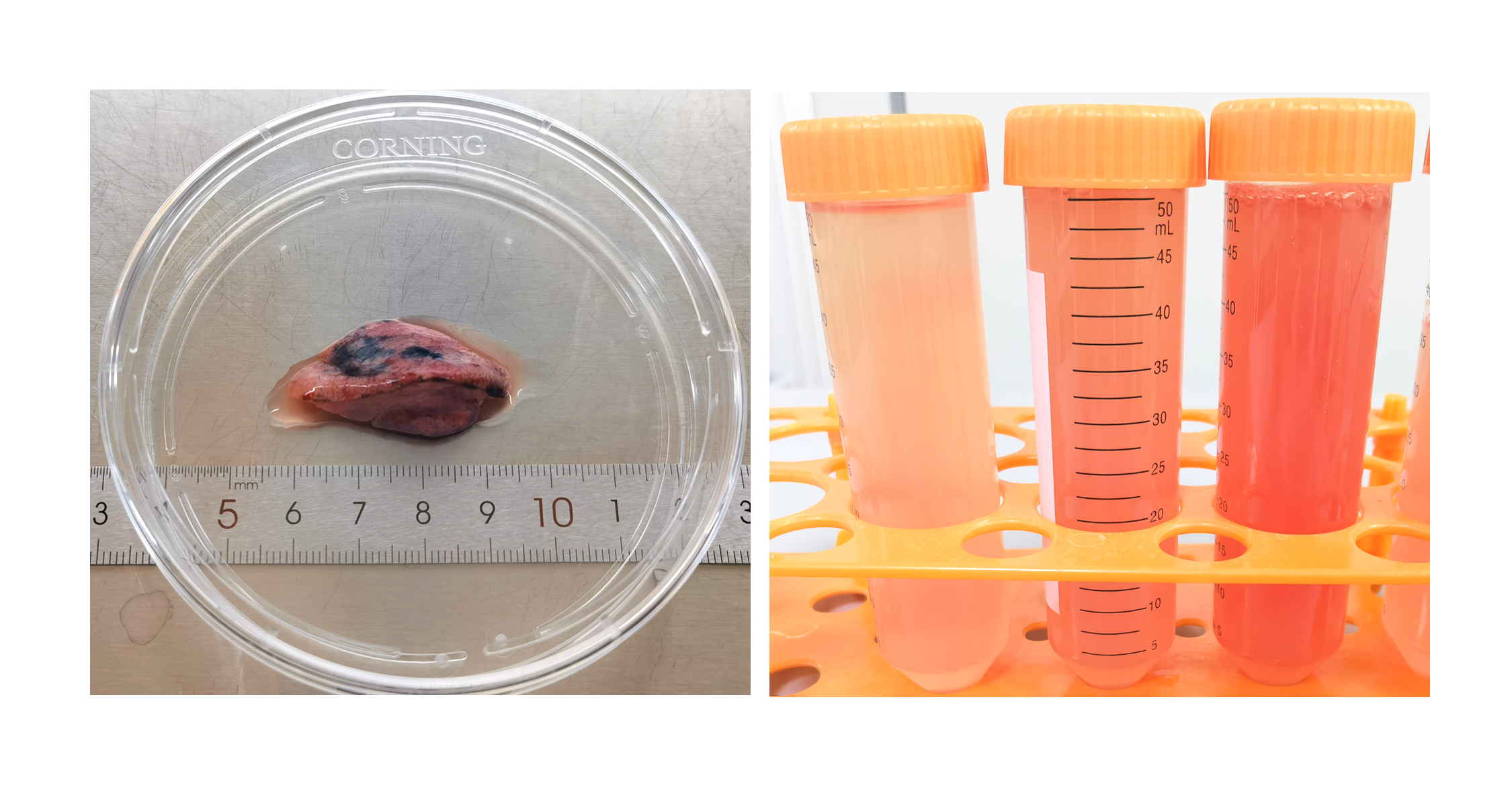

Supplement: Supplementary Figure 1 — Representative images of lung tissue from lobectomy and the collected washing fluid (n = 3 biological replicates). [file Image1.tif]

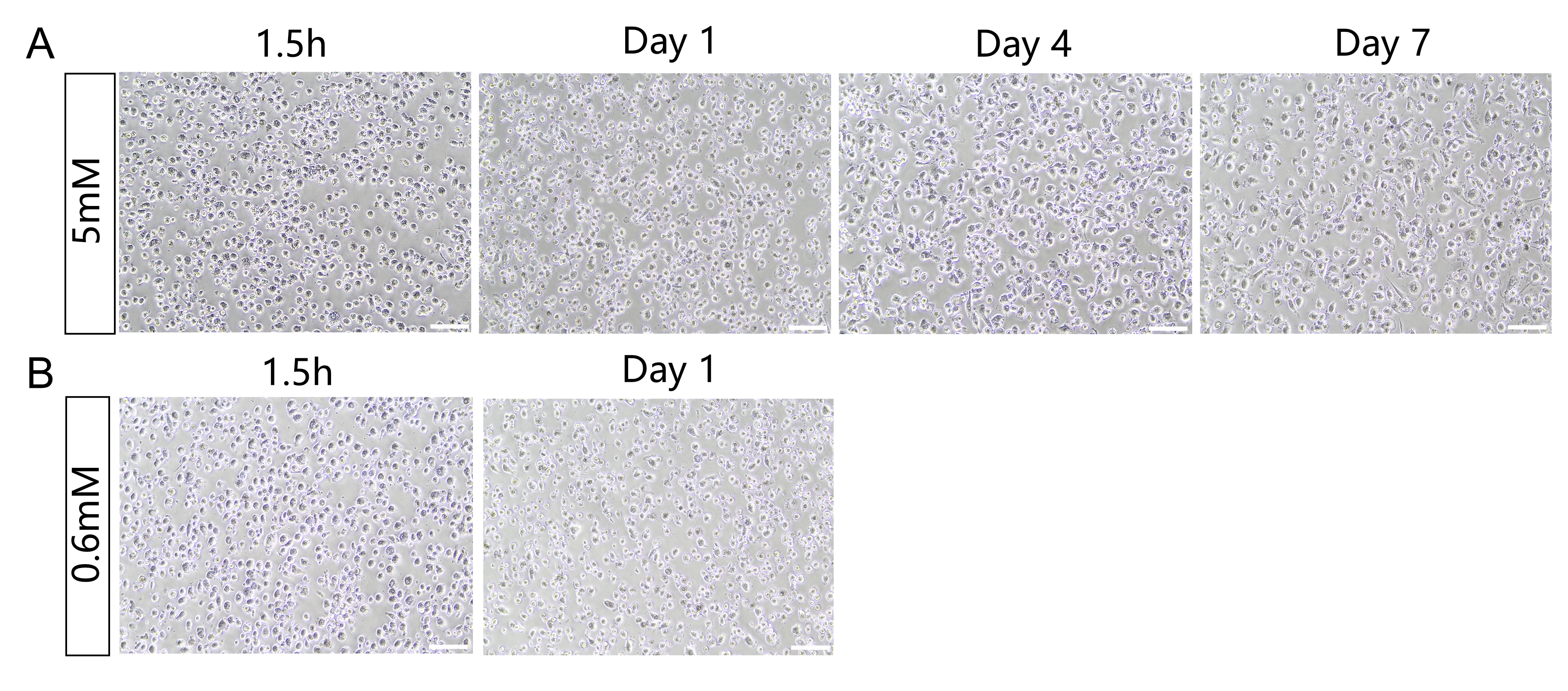

Supplement: Supplementary Figure 2 — Representative images of maintenance culture in complete medium with different glucose concentrations (n = 3 biological replicates). Scale bar, 100 μm. [file Image2.tif]

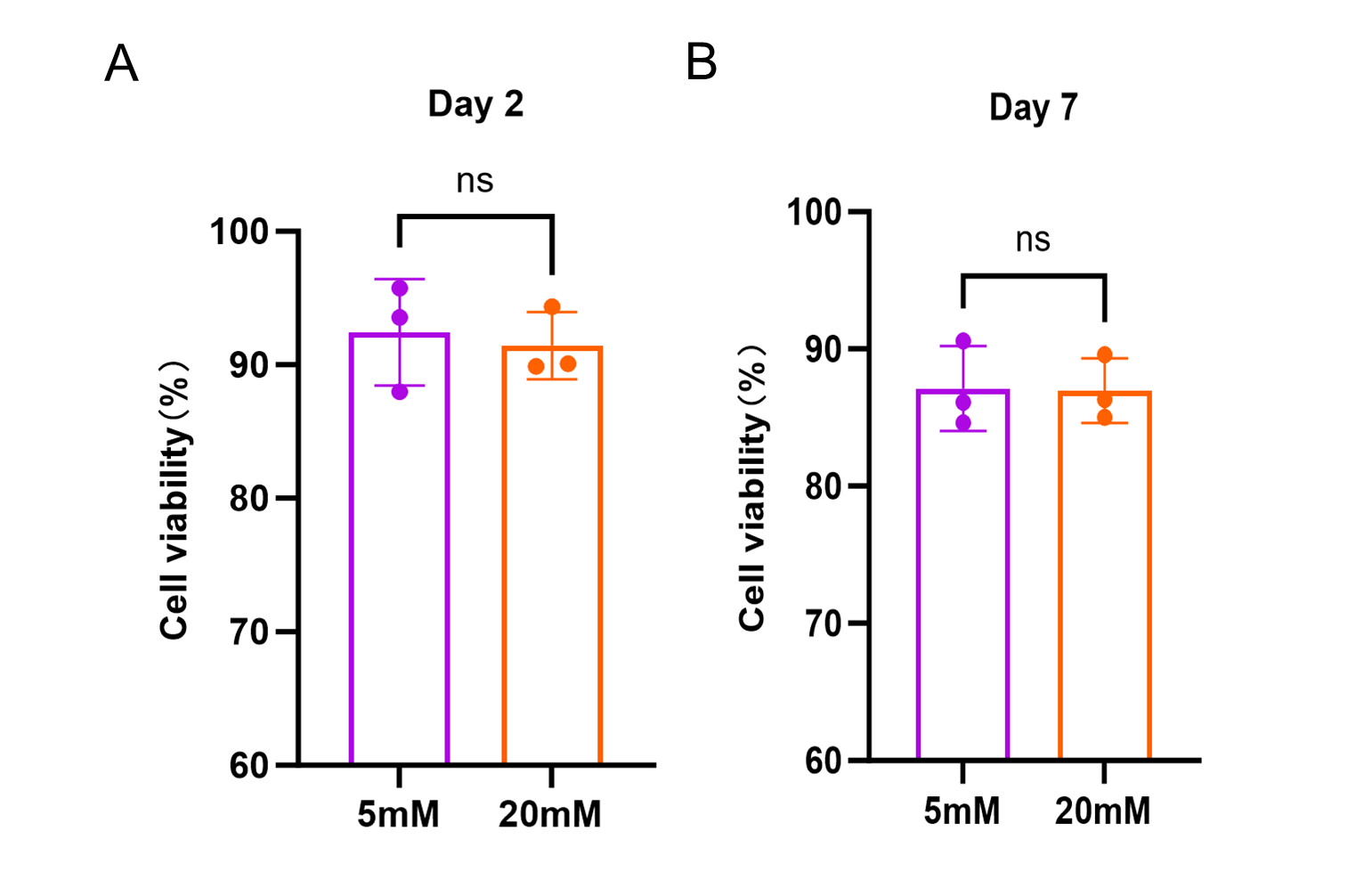

Supplement: Supplementary Figure 3 — CCK-8 assay was performed to assess AMs viability after 2 days of high glucose (20mM) exposure (A) and a subsequent 5 days resting period in normal glucose (5mM) medium (B). P-value was calculated by paired Student’s t-test. All data were presented as mean ± SD (n = 3 biological replicates with paired samples). ns, not significant. [file Image3.tif]

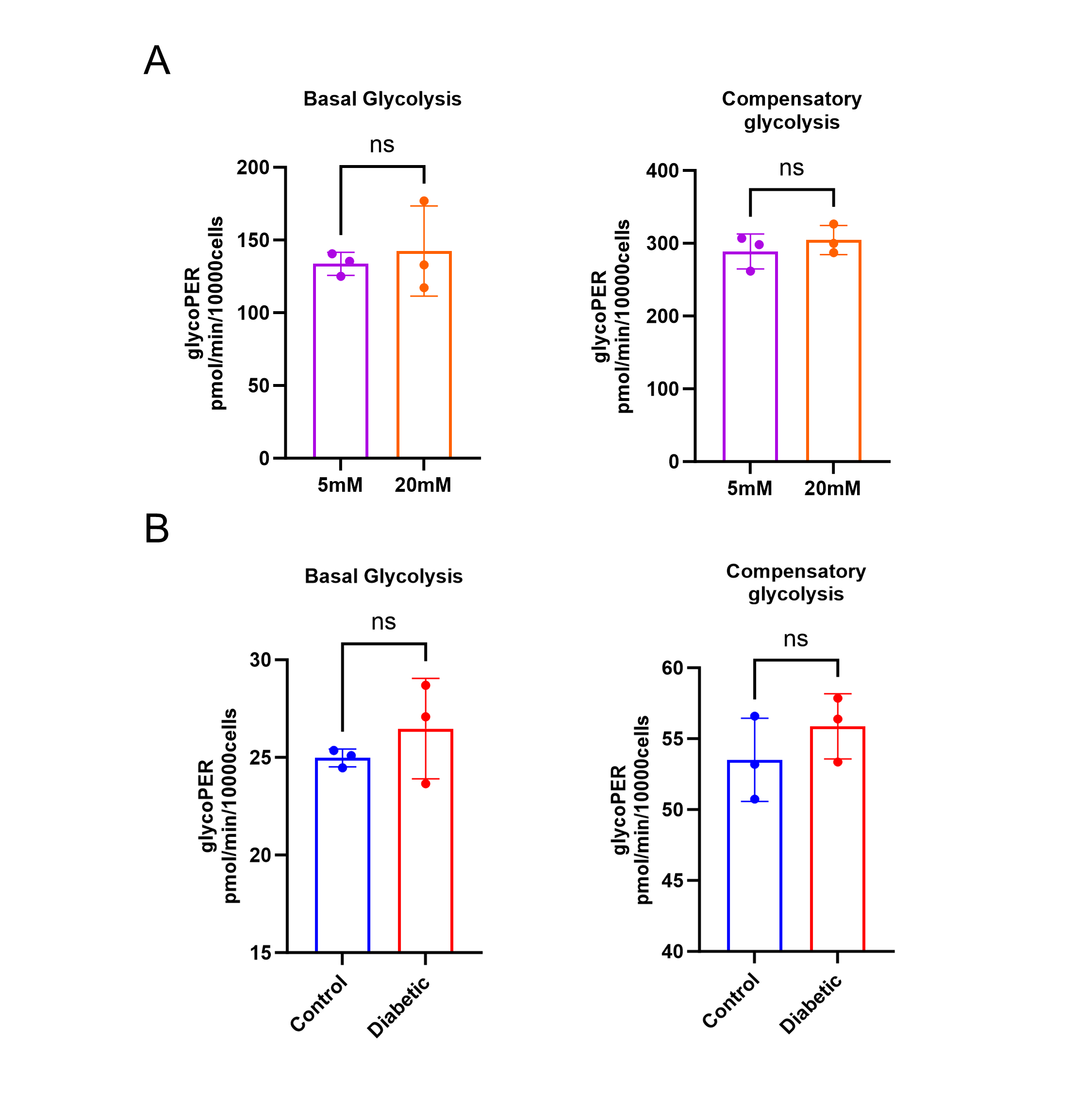

Supplement: Supplementary Figure 4 — GlycoPER measurement by Seahorse XFe96 analyzer. (A) Basal and compensatory glycolysis in immune memory model under 5mM vs 20mM glucose (n = 3 biological replicates with paired samples). (B) Basal and compensatory glycolysis in patients with or without diabetes (n = 3 biological replicates). All data were presented as mean ± SD. P-values: paired t-test (A), Mann-Whitney U test (B). ns, not significant. [file Image4.tif]

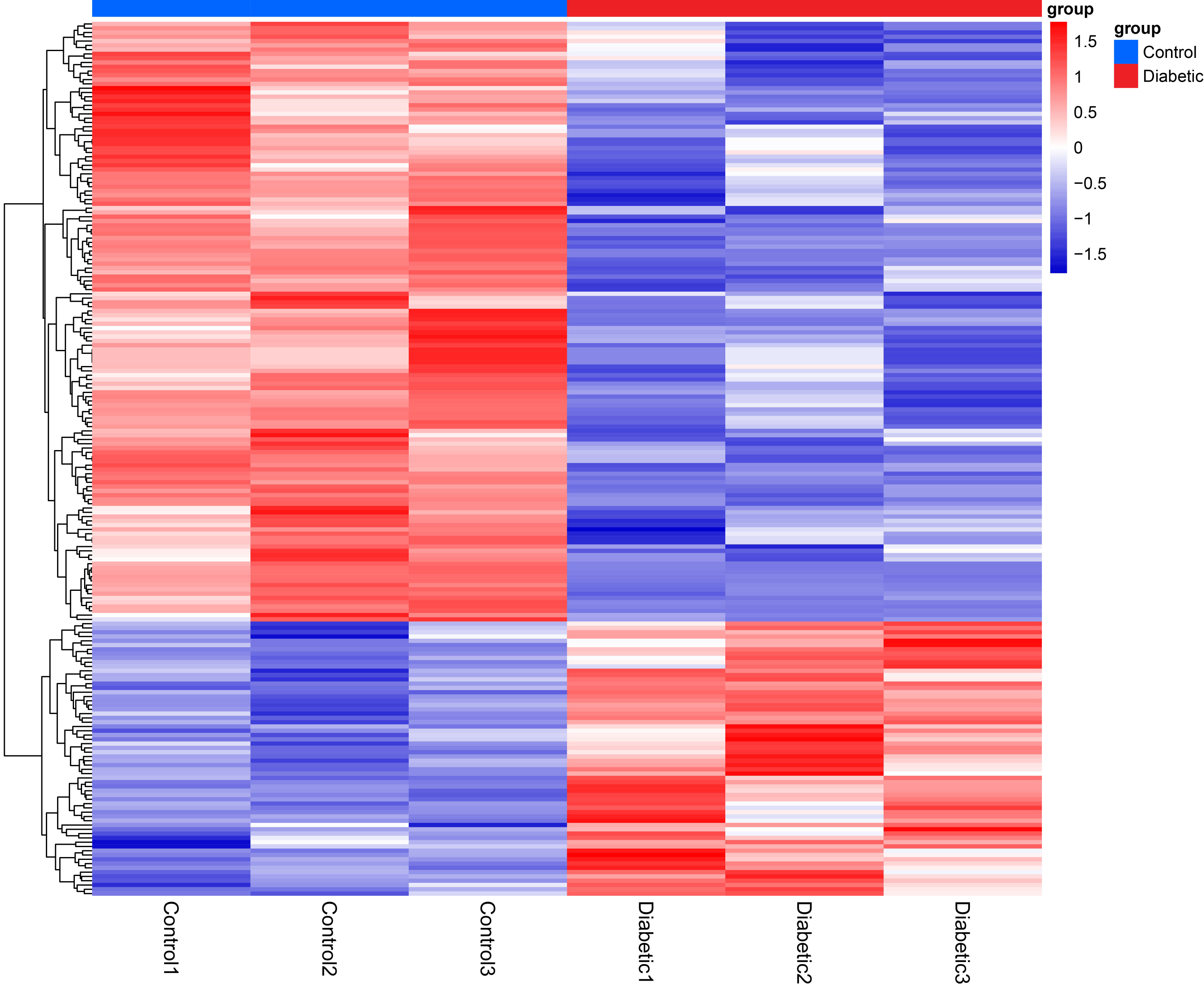

Supplement: Supplementary Figure 5 — Heatmap analysis of the differentially abundant metabolites. The significance thresholds were set at p-value < 0.05 (horizontal dashed lines) and VIP > 1 (n = 3 biological replicates). [file Image5.tif]

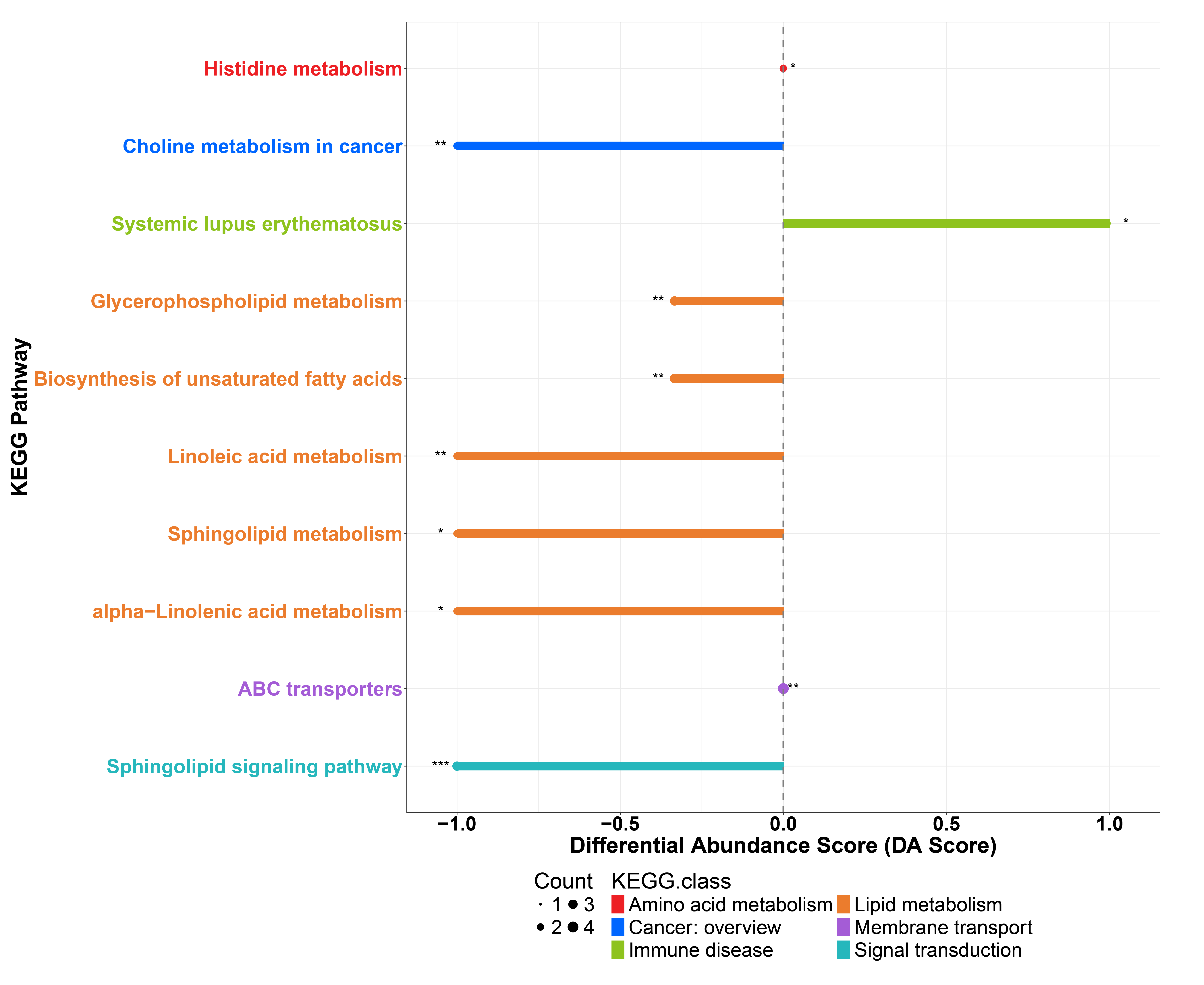

Supplement: Supplementary Figure 6 — KEGG pathway DA scoring. The X-axis represents the regulation across the entire pathway (positive values correspond to upregulation; negative values to downregulation). Bar length reflects the magnitude of the DA score. *P < 0.05, **P < 0.01, ***P < 0.001 (n = 3 biological replicates). [file Image6.tif]

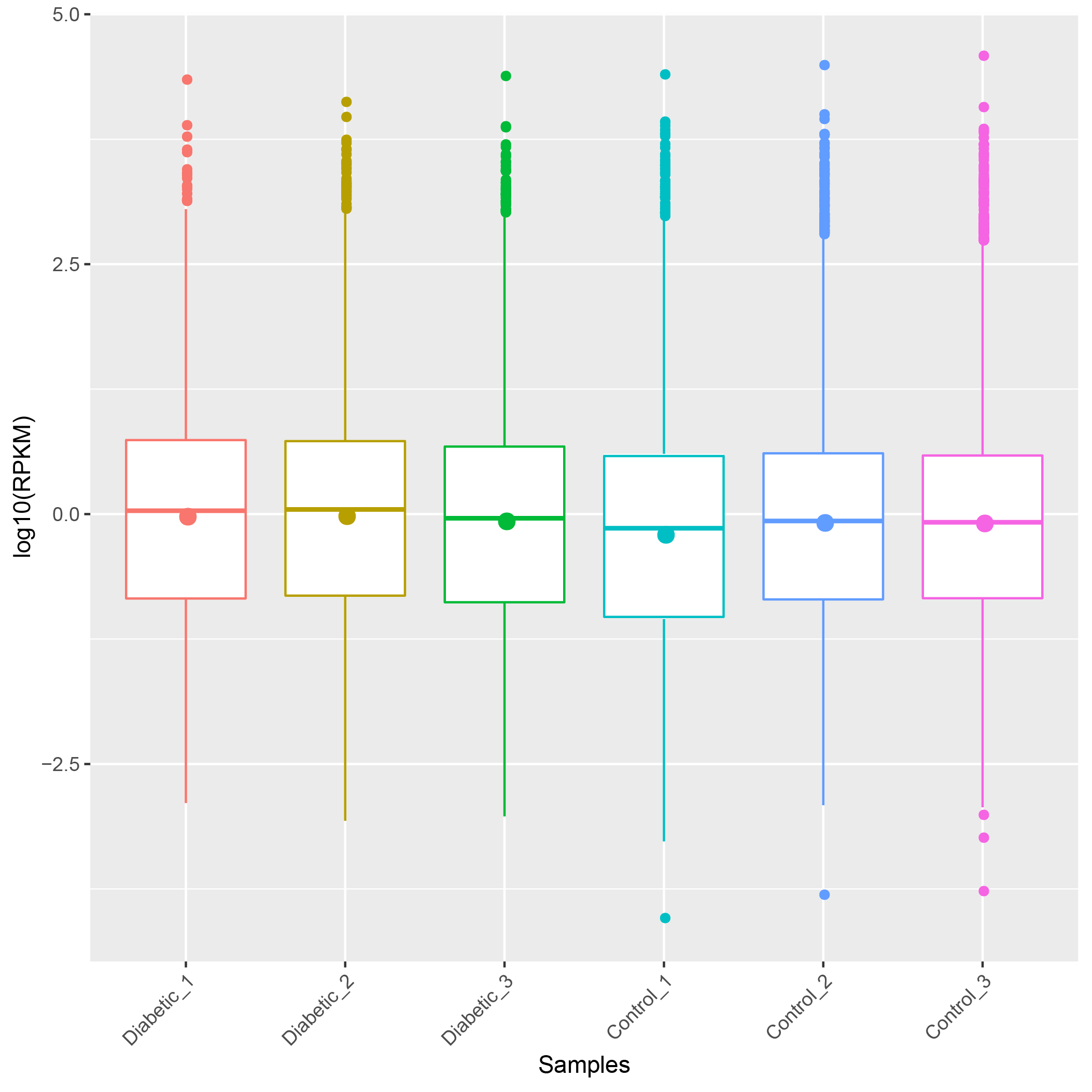

Supplement: Supplementary Figure 7 — RPKM, Reads per kilobase per million reads values were calculated for boxplot visualization of gene expression distributions across individual samples (X-axis: the sample name, Y-axis: log10 (RPKM). For each box, the center line indicates the median, box boundaries represent the upper and lower quartiles, and whiskers denote the maximum and minimum values (n = 3 biological replicates). [file Image7.tif]

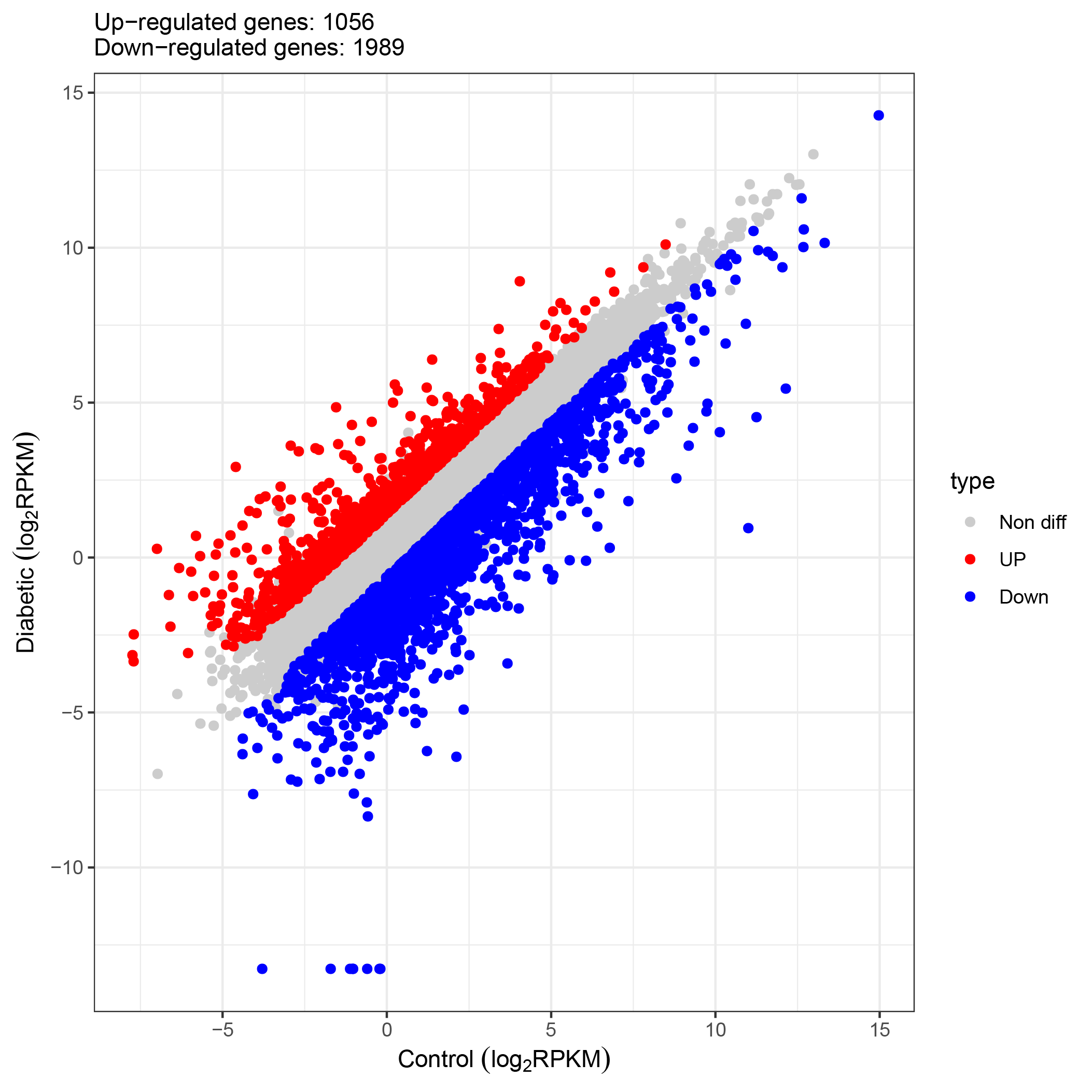

Supplement: Supplementary Figure 8 — Visualizations of gene expression levels are presented through scatter plots of RPKM values. Genes that were significantly downregulated are shown as blue dots, upregulated genes as red dots, and those with no significant expression change as gray dots (n = 3 biological replicates). [file Image8.tif]

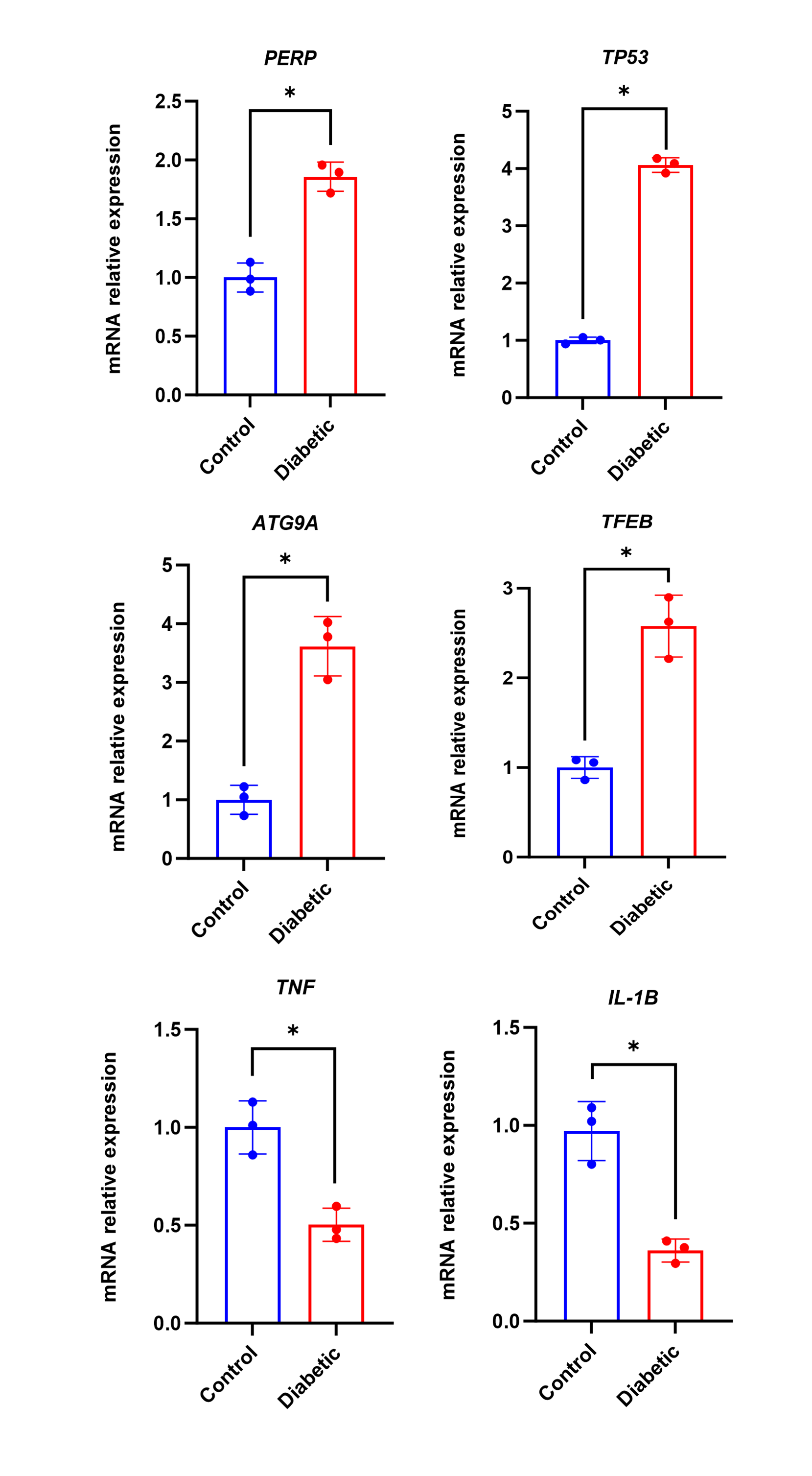

Supplement: Supplementary Figure 9 — RT-qPCR analysis of gene expression levels of PERP, TP53, ATG9A, TFEB, TNF and IL1B. P-value was calculated by unpaired Mann-Whitney U test. All data were presented as mean ± SD (n = 3 biological replicates), *P < 0.05. [file Image9.tif]

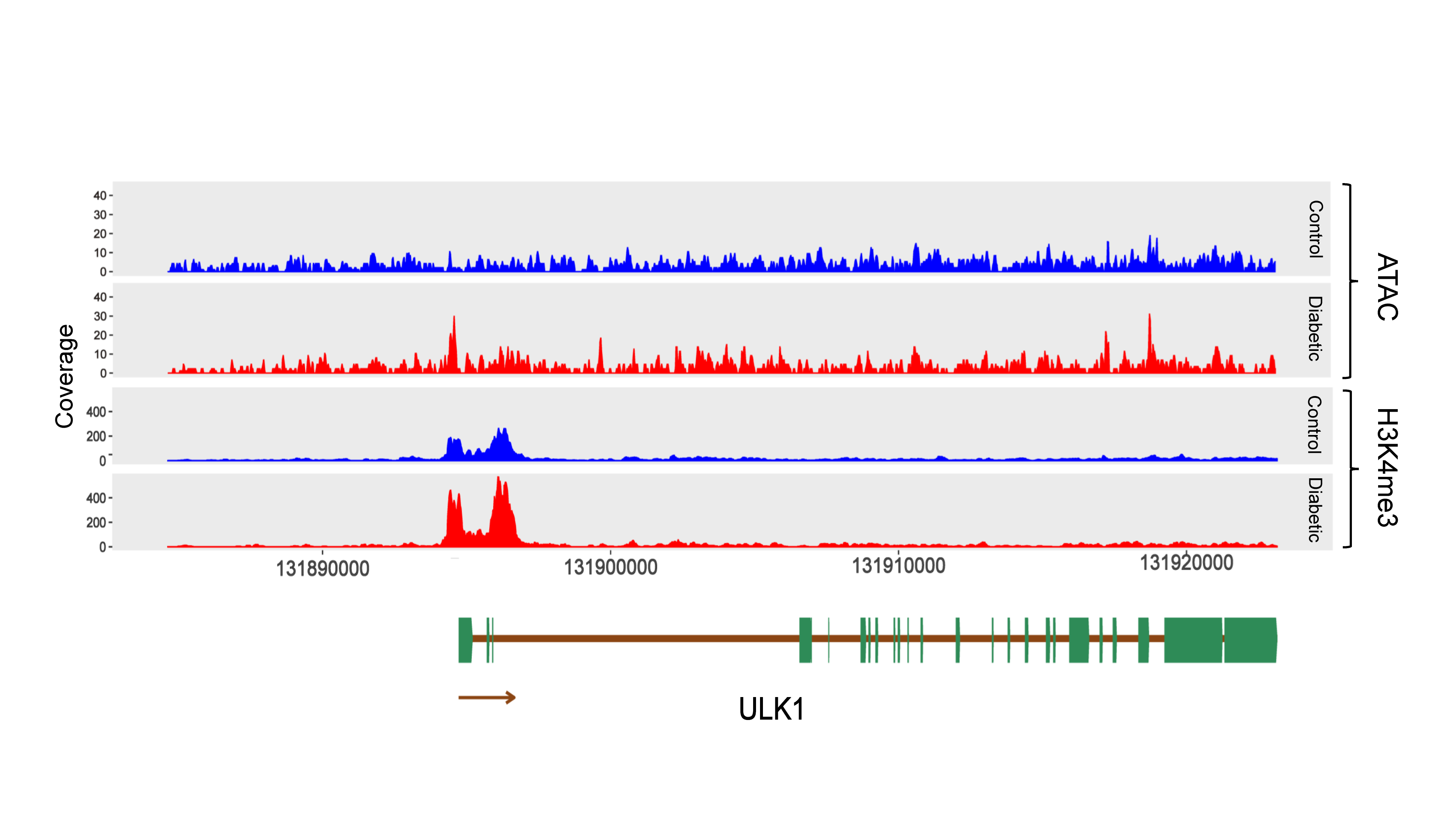

Supplement: Supplementary Figure 10 — Integrative GenePlots for ULK1 showing ATAC-seq, H3K4me3 CUT&Tag reads from representative samples of AMs between two groups (n = 3 biological replicates for ATAC-seq and n = 2 biological replicates for CUT&Tag). [file Image10.tif]

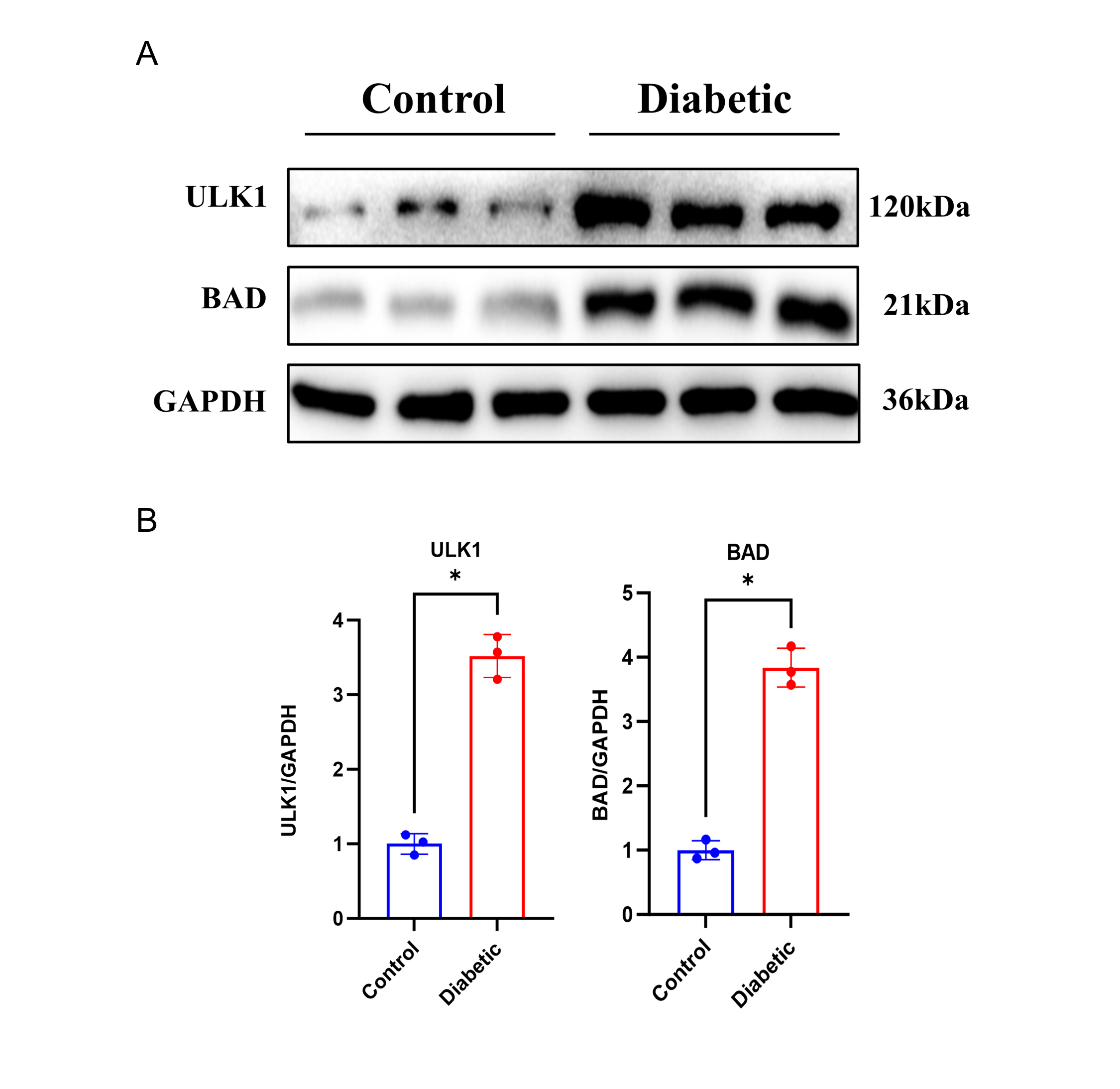

Supplement: Supplementary Figure 11 — Western blot analysis of ULK1 and BAD. P-value was calculated by unpaired Mann-Whitney U test. All data were presented as mean ± SD (n = 3 biological replicates), *P < 0.05. [file Image11.tif]
